# Stochastic, individual animal systems simulation model of beef cow–calf production: development and validation

**DOI:** 10.1093/tas/txac155

**Published:** 2022-12-03

**Authors:** Dustin G Aherin, Robert L Weaber, Dustin L Pendell, Jessica L Heier Stamm, Robert L Larson

**Affiliations:** Department of Diagnostic Medicine/Pathobiology, College of Veterinary Medicine, Kansas State University, Manhattan, KS 66506, USA; Beef Cattle Institute, College of Veterinary Medicine, Kansas State University, Manhattan, Kansas 66506, USA; Beef Cattle Institute, College of Veterinary Medicine, Kansas State University, Manhattan, Kansas 66506, USA; Department of Animal Sciences and Industry, Kansas State University, Manhattan, KS 66506, USA; Beef Cattle Institute, College of Veterinary Medicine, Kansas State University, Manhattan, Kansas 66506, USA; Department of Agricultural Economics, Kansas State University, Manhattan, KS 66506, USA; Industrial & Manufacturing Systems Engineering, Kansas State University, Manhattan, KS 66506, USA; Beef Cattle Institute, College of Veterinary Medicine, Kansas State University, Manhattan, Kansas 66506, USA; Department of Clinical Sciences, College of Veterinary Medicine, Kansas State University, Manhattan, KS 66506, USA

**Keywords:** beef cattle, cow–calf, model development, model validation, stochastic, systems model

## Abstract

A stochastic, individual animal systems simulation model describing U.S. beef cow–calf production was developed and parameterized to match typical U.S. Angus genetics under cow–calf production conditions in the Kansas Flint Hills. Model simulation results were compared to available actual, multivariate U.S. cow–calf production data reported according to beef cow–calf standardized performance analysis (**SPA**) methodology through North Dakota State University’s CHAPS program to assess model validity. Individual animal nutrition, reproduction, growth, and health characteristics, as well as production state are determined on a daily time step. Any number of days can be simulated. These capabilities allow for decision analysis and assessment of long-run outcomes of various genetic, management, and economic scenarios regarding multiple metrics simultaneously. Parameterizing the model to match Kansas Flint Hills production conditions for the years 1995 through 2018, 32 different genetic combinations for mature cow weight and peak lactation potential were simulated with 100 iterations each. Sire mature cow weight genetics ranged from 454 to 771 kg in 45 to 46 kg increments. Sire peak lactation genetics were considered at 6.8, 9, 11.3, and 13.6 kg/d for all eight mature cow weights. Utilizing model results for the years 2000 to 2018, raw model results were assessed against actual historical cow–calf production data. Exploratory factor analysis was applied to interpret the underlying factor scores of model output relative to actual cow–calf production data. Comparing modeled herd output with CHAPS herd data, median average calf weaning age, average cow age, percent pregnant per cow exposed, and percent calf mortality per calf born of model output was 3.4 d greater, 0.2 yr greater, 1 percentage point less, and 1.7 percentage points greater, respectively. Subtracting the median CHAPS pre-weaning average daily gain from the median modeled pre-weaning average daily gain for each of the eight respective mature cow weight genetics categories, and then calculating the median of the eight values, the median difference was −0.21 kg/d. Performing the same calculation for birth weight and adjusted 205 d weaning weight, the modeled data was 4.9 and 48.6 kg lighter than the CHAPS data, respectively. Management and genetic details underlying the CHAPS data were unknown.

## INTRODUCTION

In 2019, the National Academies of Sciences, Engineering, and Medicine identified the advancement of multidisciplinary systems approaches as the highest priority in order to generate critical food and agriculture breakthroughs by 2030 (NASEM, 2019). Holistic solutions to agriculture’s greatest challenges will only be discovered if the scientific community relegates single-discipline approaches in favor of multidisciplinary quantitative models (NASEM, 2019).

The time delays, complex and prolonged feedback structure, and significant capitalization inherent to the beef industry prohibit many long-term or large-scale studies. By integrating the literature from past research and knowledge from numerous specialized fields, modeling enables enhanced understanding in a time-efficient and cost-effective manner, not only by capitalizing on past discoveries, but also by identifying research gaps. Furthermore, Shafer et al. (2007) demonstrated that beef production’s natural biological variation often calls for stochastic modeling features, which can be difficult to replicate in the real world due to the previously discussed constraints.

Systems modeling often demands multidisciplinary expertise and cooperation. A multidisciplinary approach can identify emergent properties (Ebersohn, 1976; Fischer, 2008), which result from allowing discipline-specific knowledge to interact within a complex system. Single-discipline, physical experiments often provide the inputs and grounding for systems modeling, while modeling helps identify: 1) knowledge gaps where further experimentation or data collection is warranted and 2) the true contribution of new discoveries or novel strategies to a broader system.

Researchers have developed systems-based models to assess complex beef production questions (Hirooka, 2010) ranging from specific biological traits such as growth curves (Richards, 1959) to broader topics related to production system bioeconomics (Davis et al., 1994) and life-cycle assessments of environmental impacts (Asem-Hiablie et al., 2019). Tedeschi and Fox (2014) summarize the evolution of computer-based ruminant nutrition models that inform decision support systems, such as the Cornell Net Carbohydrate and Protein System (CNCPS; Fox et al., 2004) and the Cornell Value Discovery System (Tedeschi et al., 2004). Researchers at the U.S. Meat Animal Research Center also developed a model for assessing the nutrient demands of maintenance and growth, the Decision Evaluator for the Cattle Industry (DECI; Williams and Jenkins, 2003a, 2003b, 2003c). While most nutrition-focused simulation models have been aimed at assessing energy demands and energy efficiency, the breeding and genetics field has used simulation modeling and sensitivity analysis to determine the relative economic value of varying phenotypic and genetic traits (MacNeil et al., 1994, 1997). One of the most expansive beef production models is the Colorado Beef Cattle Production Model (CBCPM; Bourdon and Brinks, 1987; Shafer et al., 2005), an adaptation of the foundational Texas A&M University (TAMU) model (Kahn and Spedding, 1983; Sanders and Cartwright, 1979). CBCPM is a broad-scope, whole-herd model that accounts for both genetics and nutrition in combination with forage production and economics.

Despite its many benefits, systems modeling’s application to the beef industry, and agriculture in general, is far from reaching its potential. NASEM (2019; 2) identified three primary goals for food and agriculture to achieve by 2030: “1) improving the efficiency of food and agricultural systems, 2) increasing the sustainability of agriculture, and 3) increasing the resiliency of agricultural systems to adapt to rapid changes and extreme conditions.” For reasons already discussed, NASEM (2019) strongly recommends prioritizing multidisciplinary systems techniques to achieve the outlined goals.

Past models and their derivative equations require continuous updates based on research advances and production data availability (Hirooka, 2010). Models make it possible to assess the impact of discipline-specific advances and their fit in the broader production system. There are several more reasons to recreate or update beef production models. Growing computing power creates the possibility for more model complexity without sacrificing computing time. In addition, building models in modern, open-source programming languages encourage application. Perhaps most importantly, model building provides the modelers with synthesized learning about how an entire system functions.

Verification establishes that a model performs conceptually and mathematically as intended by the modeler; whereas validation assesses how accurately a model characterizes reality (Thacker et al., 2004). Often, model development is motivated by a shortage of actual data describing the scope of interest in a particular system. Coupling data scarcity with the ambiguity of appropriate methodology, quantitative validation has chronically challenged modelers (Barlas, 1996).

As described by Shafer et al. (2005), quality, long-run datasets with defined whole-system parameters may be unavailable in the beef industry. For a short-duration evaluation, Villalba et al. (2006) controlled actual environment and management as closely as possible to match model parameters and then compared between real and simulated data to establish model validity. Unfortunately, there was no ability to assess long-run feedback structures and compounding of differences.

Many have demonstrated the flaws in applying inferential statistics to compare computer-simulated data to actual data (Harrison, 1990; Hofmann et al., 2018). Conversely, sensitivity analysis, often part of model validation, refers to multiple qualitative and quantitative methodologies aimed at evaluating model output response to alternative model parameters (Frey and Patil, 2002; Iooss and Lemaitre, 2015; Trucano et al., 2006; Walker and Fox-Rushby, 2001). Xiao et al. (2017) describe principal component analysis (**PCA**) methods to address multivariate output sensitivity analysis in complex models.

Exploratory factor analysis (**EFA**) interprets multivariate patterns and interrelationships by estimating an underlying, unobservable (latent) factor structure that influences observed data values (Luo et al., 2019; Park et al., 2002). Whereas PCA utilizes total variation among variables without consideration for its source, EFA separates variable variation into common variance and unique variance (Park et al., 2002). For each measured variable, common variance represents the variance explained by one or more latent factors, whereas unique variance stems from other sources, including measurement error (Luo et al., 2019; Park et al., 2002).

### Objective

The present research objective was the development of a stochastic systems simulation model on an individual-based, daily time-step capable of accounting for the nutrition, reproduction, genetics, health, and economic conditions relevant to cow–calf production. The use of EFA to assess model validity in circumstances where actual outcome data (e.g., weaning weight, reproductive rate, etc.) cannot be paired with respective input parameters (e.g., management, genetics, nutrition) was also investigated.

## MATERIALS AND METHODS

### Model Design and Simulation for Validation

Written in R statistical software (R Core Team, 2019), the model represents a cow–calf production system by determining animal characteristics, production phase, and production state at the individual level on a daily time step. Animal characteristics determined daily include age, weight, BCS, lactation, nutrition requirements, nutrient availability, reproductive status, morbidity, and mortality. The daily outcomes of these traits are determined by interactions between the animal’s genetics, the previous day’s trait outcomes, variable stochasticity, the animal’s production phase and state, calendar date, and the application of general decision rules. Linear production phases include nursing calf, post-weaning non-pregnant replacement heifer, bred replacement heifer, 2-yr-old cow, 3-yr-old cow, and mature cow (≥4-yr-old). Recurring production states include lactating and non-lactating, cycling and non-cycling, and pregnant and non-pregnant. General decision rules are parameterized to meet a defined set of management goals. Simulated management decisions such as culling, replacement, feed source, and exposure for breeding are made daily contingent on the calendar date and feedback provided by animal characteristics at the individual and aggregate herd levels.

Model parameterization is flexible and subject to the specific scenario of interest. Any length of time and any number of production cycles can be simulated, generating a chain-of-events dependent on both past and present conditions. Parameters for the present exercise are described in subsequent paragraphs and listed in detail in Supplementary Table S1.

Given the goal of the present work and the environment’s importance to cow–calf production, parameters for temperature, precipitation, grazed forage nutrient content, and stocking rate ultimately influences nutrition requirements (temperature) and grazed nutrient availability (precipitation, grazed forage nutrient content, and stocking rate). The availability of forage production data under Kansas Flint Hills grazing conditions made that region an ideal environment to simulate. Angus is the most prevalent breed in the U.S. commercial cow herd, thus typical Angus genetics was used as a representation of U.S. commercial beef cattle genetics.

Simulated sire mature cow weight (**MW**) genetic potential (**GP**) ranged from 454 to 771 kg in 45 to 46 kg increments with sire peak lactation (**PL**) GP set to 6.8, 9, 11.3, or 13.6 kg/d. In total, 32 combinations (8 Sire MW GPs and 4 Sire PL GPs) with varying MW and PL genetics were simulated.

The model is parameterized for a goal of 100 breeding females exposed during the breeding season and each scenario herd is simulated 100 iterations. Each iteration runs for 24 production years (1995 to 2018) with historical inputs as specific as possible to the Flint Hills region for precipitation and temperature. A “production year,” as defined in the model, is the time from calving in year *i* to either calving in year *i*+1 (calving interval) or culling for each individual breeding female. Individual breeding females are removed from the herd between calving and the start of the breeding season if their calf dies within the said time frame or at any day corresponding with the female’s own mortality. Further culling occurs at weaning or pregnancy diagnosis, whichever is later, according to rules outlined in General Decision Rules. Thus, the length of each production year will vary between individual animals. The 5 yr 1995 through 1999 are used as burn-in years to allow for model stabilization following the exogenous parameter inputs in the initial year (1995) and are not reported in the results.

After 100 iterations of the 590 kg MW—11.3 kg/d PL scenario, the 95% confidence interval for mean kg weaned per cow exposed, which incorporates fertility, health, nutrition, and growth, was calculated for the final year of the simulation (2018). The 95% confidence interval for mean kg weaned per cow exposed was 175.72 ± 1.45 kg per cow exposed. Given the small 95% confidence interval and a greater than 12-h run time for a 100-iteration simulation, it was deemed that 100 iterations for each scenario were sufficient.

To determine the number of burn-in production years, within-herd cow age distribution was assessed. The mean herd proportion for a given cow age across production years only changed at the third decimal place when progressively excluding the production year 1995; production years 1995 and 1996; production years 1995, 1996, and 1997; production years 1995, 1996, 1997, and 1998; and production years 1995, 1996, 1997, 1998, and 1999. Visual inspection also showed that cow herd age distribution at model initialization was well within the range of subsequent endogenous production year results. A 5-yr burn-in period (1995 to 1999) thus was deemed sufficient.

### General Decision Rules

The model goal is to expose 100 females for breeding between May 1 and July 3 (63 d) each year. Heifer and cow breeding season start and end dates are identical. Pregnancy status is determined 60 d after the end of the breeding season. All non-pregnant females are sold at calf weaning. In addition, minimum culling levels are set for each age group (Supplementary Table S1.12). If the percent non-pregnant within an age group has not reached the minimum level, voluntary culling (assumed to result from disposition, foot quality, udder quality, etc.) occurs until the minimum for each specific age group is reached. All 13-yr-old cows at the time of pregnancy detection are culled on the cull date. Any female that aborts a pregnancy after the cull date is culled on that day. Heifer calves are kept to replace the culled females, plus an addition calculated from past cow losses (mortality, abortion, calf-loss before breeding season) that occur between weaning and the next breeding season. Replacement heifers are selected in order from oldest to youngest. If replacement heifer requirements exceed the number of raised heifers, non-pregnant replacement heifers are purchased with traits matching the raised heifer population.

All calves are sold on the weaning date and weaned on the same date. The weaning date is set as the date upon which the oldest calf is 220 d old.

Bulls are not accounted for in the model. Because the goal of each scenario is to expose the same number of females for breeding, it assumed any cost differences from bulls would be minor.

Within the model, the grazing season spans from May 1 to October 31 (KSU and KDA, 2019). McMurry (2009) estimated average U.S. cow size at 612.25 kg (1,350 pounds), while the CHAPS database reported a 629.9 kg (1,389 pound) benchmark cow weight for 2018 (CHAPS, 2018). The average full-season cow–calf pair acreage allocation in the Flint Hills between 1995 and 2019 was 7.3 acres per pair (Dhuyvetter et al., 2009; KSU and KDA, 2017, 2019). Assuming a 1,350 pound (612.25 kg) average cow weight between 1995 and 2019, the metabolic weight (weight^0.75) of a 612.25 kg (1,350) pound cow was indexed as 1. For each scenario, 7.3 acres per pair allocation was changed according to the metabolic weight of the mean mature cow weight as a percentage of the metabolic weight of a 1,350-pound cow (Supplementary Table S1.5). The same acreage allocation adjustments were made for full-season yearling heifer grazing and post-weaning grazing of replacement heifer calves from their base acreage allocations of 4 acres per head and 2.7 acres per head (Dhuyvetter et al., 2009; KSU and KDA, 2017, 2019), respectively (Supplementary Table S1.5).

### Genetics

Of 48,058 total sale lots from 164 video auctions occurring from 2010 through 2016, McCabe et al. (2019) reported that 34% of steer calf lots and 31% of heifer calf lots were described as Angus sired with no Brahman influence. In a recent popular press survey, 53% of respondents reported a predominantly Angus cow herd and 55% most recently purchased an Angus bull (Ishmael, 2020). The responses were not weighted for cow herd size. The simulated cow herd is assumed to have 100% Angus genetic makeup.

An animal’s GP for weaning weight (WW) is a base value determined from regression on the animal’s MW GP summed with its estimated breeding value (EBV) for WW. An animal’s GP for birth weight (BW) is determined by a similar procedure, but through regression on WW GP. This process results in individuals with correlated GPs across the traits of interest reflective of their respective MW GP. Within each MW and PL category, the GP difference for any trait is equivalent to an EBV difference. The MW GP for individual animals within the simulation is drawn from a normal distribution with the mean determined by averaging the sire and dam MW GPs for each simulated mating. Thus, each mating potentially generates a distribution with a unique mean. The standard deviation (SD) is set to 25.4 kg (56 pounds) for each of the eight MW category GP distributions. The SD was equivalent to the possible change value of an EBV with BIF accuracy of 0.3 (AAA, 2019a). Parents were assumed to have known genetic values with a BIF accuracy of 0.99 (BIF, 2010). Progeny genetic potentials were the sum of the mean parental values and a randomly sampled EBV with mean zero and SD as described above. Within the model, MW GP is defined as mature cow weight at a body fat composition of 0.1889, body condition score (BCS) of 5 on a 1 to 9 scale (NASEM, 2016). Sire MW GP is static at the scenario-defined level. Dam MW GP is her actual MW GP as determined in the stochastic method presently described.

The preliminary PL GP for each individual animal is determined in the same manner as MW GP, except that the SD for the GP normal distribution is arbitrarily set to 1 kg (2.205 pounds) as in reality, no genetic evaluations calculate an EPD for PL. Sire PL GP is static at the scenario-defined level and dam PL GP determination follows the same method as dam MW GP.

Historical EPD trends by birth year for BW and WW were gathered from the American Angus Association and then converted (multiply by two) to EBVs to account for genetic trends across the simulated time period (AAA, 2019b; Supplementary Table S1.2). Although an increasing MW trend was also present (AAA, 2019b), it was assumed that the same BW and WW genetic trend occurred even with fixed sire MW GP. This can be interpreted as a breeding objective in which MW is held constant while maintaining genetic improvement for BW and WW consistent with breed average. For any year *i*, Sire EBVs for BW and WW are the average EBVs for year *i*−2 through year *i*−5, assuming a sire is used four mating seasons and his first progeny are born when he is 2-yr-old. The sire EBV for WW and BW were then converted to a GP base representative of actual weaning weights, as follows, to simplify intra-model calculations.

#### Sire WW GP


$$\begin{array}{ll} & Sire\;\;\;WW\;\;\;GP\;\;\;Base=0.42*1400\\ & +\left(Sire\;\;\;MW\;\;\;GP*2.205-1400\right)*0.125, \end{array}$$ (1)

where Sire WW GP Base and Sire MW GP are in pounds.

Therefore,


$$\begin{array}{ll} & Sire\;\;\;WW\;\;\;GP_{i}=Sire\;\;\;WW\;\;\;GP\;\;\;Base\\ & +Average\;\;\;Sire\;\;\;WW\;\;\;EBV_{i}, \end{array}$$ (2)

where Sire WW GP_*i*_ (pounds) is the Sire WW genetic potential for calves born in year *i*.


$$\begin{array}{ll} & Average\;\;\;Sire\;\;\;WW\;\;\;EBV_{i}\\ & =(\Sigma_{i-2}^{i-5}AAA\;\;\;WW\;\;\;EBV_{i})/4, \end{array}$$ (3)

where AAA WW EBV_*i*_ (pounds) is the average WW EBV for Angus calves born in year *i* (AAA, 2019b).

Critical to the WW GP conversion is the assumption that a 100 kg deviation from 635 kg MW base causes a 12.5 kg directionally similar change in base WW GP (Doye and Lalman, 2011). Referencing actual calf weaning weight as percent of MW from Doye and Lalman (2011), Scasta et al. (2015), and Ramsay et al. (2017), 42% of 1,400 pounds (635 kg) was used as a constant in the Sire WW GP Base equation. Applying the Sire WW GP Base and the Average Sire WW EBV for 1995 facilitates the assumption that calves born in 1995 and reported in Ramsay et al. (2017) had an adjusted 205 d weight equal to their genetic potential.

Sire BW GP Base was determined by regressing Adjusted BW on Adjusted WW (assuming 205 d adjusted WW was equal to genetic potential) using 2017- and 2018-born bull calf records from 424 Gelbvieh, Simmental, Red Angus, and respective Angus crosses (EPR, 2019) after converting bull WW to steer WW by a factor of 0.97 (Lee et al., 1997). The original constant of 60.202 was reduced to 54.202 to convert to an Angus base considering 2018 across breed BW EPD adjustment factors after converting to EBV units (Kuehn, 2018).

#### Sire BW GP


$$\begin{array}{ll} & Sire\;\;\;BW\;\;\;GP\;\;\;Base=0.037*\\ & \left(Sire\;\;\;WW\;\;\;GP\;\;\;Base+Sire\;\;\;WW\;\;\;EBV_{1992}\right)\\ & +54.202. \end{array}$$ (4)

Therefore,


$$\begin{array}{ll} & Sire\;\;\;BW\;\;\;GP_{i}=Sire\;\;\;BW\;\;\;GP\;\;\;Base\\ & +Average\;\;\;Sire\;\;\;BW\;\;\;EBV_{i}, \end{array}$$ (5)

where Sire BW GP_*i*_ (pounds) is the Sire BW GP for calves born in year *i*.


$$\begin{array}{ll} & Average\;\;\;Sire\;\;\;BW\;\;\;EBV_{i}\\ & =(\Sigma_{i-2}^{i-5}AAA\;\;\;BW\;\;\;EBV_{i})/4, \end{array}$$ (6)

where AAA BW EBV_*i*_ (pounds) is the average BW EBV for Angus calves born in year *i* (AAA, 2019b).

Both WW and BW bases are on a male base. All GP mating calculations are based on steer WW GPs and bull BW GPs. Appropriate conversions to female phenotypes are described where relevant.

The preliminary BW and WW GPs for all breeding females in the initial year (1995) are calculated using the parental average GP distribution technique previously described, with the assumption that both parents have the average GPs for calves born in the years 1989 to 1992. The SD for the BW GP distribution is 3.76 pounds (1.71 kg) and the SD for the WW GP distribution is 22 pounds (10 kg) with both GPs at a 0.3 BIF accuracy based on 0.99 parental EPD BIF Accuracy (AAA, 2019a; BIF, 2010). Preliminary BW and WW GPs for subsequent generations are calculated using the average sire GP for that year’s calf crop, the dam’s individual GP, and the stochastic process described previously.

Final PL, WW, and BW GPs are determined after accounting for intra-population genetic correlations between PL and MW (+0.14; Morris and Wilton, 1976); WW and MW (+0.44; AAA, 2019c); and between BW and WW (+0.29; AAA, 2019c) by incorporating Cholesky decomposition techniques into the model (Hofert, 2013). Applying Cholesky decomposition, individual PL GPs were adjusted while individual MW GPs were held constant until the intra-population genetic correlation for a simulated calf crop was +0.14. The same procedure was applied to WW and MW GPs to achieve a +0.44 intra-population correlation. When applying Cholesky decomposition to BW and WW GPs, WW was held constant while individual BW GPs were adjusted to reach +0.29 intra-population correlation.

Assuming no heterosis in purebred Angus genetics, a BW heritability of 0.46 (AAA, 2019c) implies that 46% of the population’s phenotypic variance stems from genetic variance. Individual phenotypic birth weight was modeled from a normal distribution with the individual’s BW GP as the mean and 4.07 pounds (1.85 kg) as the SD. Phenotypic BW was further adjusted by dam age (BIF, 2010) and calf sex. Female birth weight phenotypes are 94% of their male equivalents (BIF, 2010; USDA, 2010). Original 205 d WW GPs are on a steer base with female WW GPs at 93% (Lee et al., 1997) of their steer equivalents. Adjustments to WW GP based on calf sex are made before calculating genetic pre-weaning average daily gain (ADG). Base pre-weaning ADG (BADG) potential for calf *j* is calculated with the following equation (BIF, 2010):


$$\begin{array}{l} BADG_{j}=\frac{\left[\left(WW\;\;\;GP_{j}-WW\;\;\;Dam\;\;\;Age\;\;\;Adjustment_{j}\right)-\left(BW\;\;\;GP_{j}-BW\;\;\;Dam\;\;\;Age\;Adjustment_{j}\right)\right]}{205}, \end{array}$$ (7)

where WW GP_*j*_ is the 205 d WW GP for calf *j*, adjusted for calf sex, WW Dam Age Adjustment_*j*_ is the BIF (2010) WW adjustment based on dam age and sex of calf *j*, BW GP_*j*_ is the 205 d BW GP for calf *j*, adjusted for calf sex, and BW Dam Age Adjustment_*j*_ is the BIF (2010) WW adjustment based on dam age and sex of calf *j*.

Thus, base potential for actual WW (BAWW) for calf *j* can be calculated as follows:


$$\begin{array}{l} BAWW_{j}=Actual\;\;\;BW_{j}+BADG_{j}*Weaning\;\;\;Age_{j}, \end{array}$$ (8)

where Weaning Age is the calf *j* age in *d* at weaning.


$$\begin{array}{l} Actual\;\;\;BW_{j}=rnorm(BW\;\;\;EBV_{j},4.07)-BW\;\;\;Dam\;\;\;Age\;\;\;Adjustment_{j}, \end{array}$$ (9)

where rnorm(BW GP_*j*_, 4.07) is a randomly drawn value for calf *j* from a normal distribution with mean equal to calf *j*’s BW GP and standard deviation equal to 4.07 pounds, as previously described.

Actual weaning weight is a function of calf weaning age and pre-weaning ADG subject to nutrient availability, and nutrient requirements according to NASEM (2016) equations. Prior to d 80 postpartum, there is no limit on calf ADG. Calves grow according to NASEM (2016) equations using whatever nutrients are available. At d 50, assuming a functional rumen, calves are allowed to eat forage, generally following Tedeschi and Fox (2009). At d 80, calf growth is capped to the calculated ADG need to reach their genetic potential. For calf age ≥ 80 d:


$$\begin{array}{l} Max\;\;\;ADG_{jd}=\frac{BAWW_{j}-\;\;\;Calf\;\;\;Weight_{jd}}{Weaning\;\;\;Age_{j}-d}, \end{array}$$ (10)

where Max ADG_*jd*_ is the maximum ADG allowed for calf *j* on day *d* of age and Calf Weight_*jd*_ is the calf *j* body weight on day *d* of age.

Such growth rules generate a similar pre-weaning growth as presented in Tedeschi and Fox (2009). Ultimately, calves are not allowed to outgrow their base potential for actual weaning weight.

### Nutrition

Individual, daily nutrient requirements and intake of all cows and calves are calculated using NASEM (2016) equations for net energy for maintenance (NEm) and net energy for gain (NEg). Weight, temperature, growth, gestation, and lactation are all accounted for depending on the individual’s production phase. Monthly average temperature data for Manhattan, KS (HPRCC, 2019) is included in the base maintenance NEm equation. Although not likely in real-world production, nutrition within the model is optimized by assuming individual management.

Daily net energy requirement for maintenance, gestation, and lactation (NEmR) for all animals, regardless of age is calculated as:


$$\begin{array}{l} NEmR_{jd}=\left[0.0007*\left(20-Temp_{mi}\right)+0.077\right]*CSB{W_{jd}}^{0.75}+NEmLG_{jd}, \end{array}$$ (11)

where NEmR_*jd*_ is the NEmR (megacalories, Mcal) for animal *j* on day *d*, Temp_*mi*_ is the average temperature during month *m* and production year *i*, CSBW_*jd*_ is the shrunk body weight of animal *j* on day *d*, and NEmLG_*jd*_ is the net energy (Mcal) requirement for lactation and gestation for animal *j* on day *d*.


$$\begin{array}{l} NEmLG_{jd}=NEmL_{jd}+NEmG_{jd}, \end{array}$$ (12)

where


$$\begin{array}{l} NEmL_{jd}=0.72*Lact_{d}, \end{array}$$ (13)

where


$$\begin{array}{l} Lact_{d}=\;\frac{n}{a*e^{k*n}}*AF, \end{array}$$ (14)

Lact_*d*_ is the daily lactation in kg/d, *n* is the *d* postpartum rounded to the nearest integer week, *k* = 0.1175, AF = 0.74 if cow year of age ≤2, 0.88 if cow year of age = 3, or 1 if cow year of age = 1, and


$$\begin{array}{l} a=\;\frac{1}{PL*k*e}. \end{array}$$ (15)

NEmG_*jd*_ is calculated as follows:


$$\begin{array}{l} NEmG_{jd}=km*MEpreg_{t}, \end{array}$$ (16)

where *km* = 0.576.


$$\begin{array}{l} MEpreg_{t}=\;\frac{NEpreg_{t}}{0.13}, \end{array}$$ (17)

where


$$\begin{array}{l} NEpreg_{t}=\frac{\left[CBW_{j}*\left(0.05855-0.0000996*t\right)*e^{0.03233*t-0.0000275*t^{2}}\right]}{1000}, \end{array}$$ (18)

CBW_*j*_ is the actual birth weight of calf *j*, and *t* is the day of gestation.

If a cow’s PL is less than 7 kg/d, NEmR is reduced by 12% (Montano-Bermudez et al., 1990). If a calf’s dam has a PL less than 7 kg/d, NEmR is reduced by 11% (Montano-Bermudez et al., 1990).

Daily dry matter intake (DMI; kg/d) for cows ≥2-yr-old is calculated as (NASEM, 2016):

If *t* ≤ 93, then


$$\begin{array}{l} DMI_{d}=CSBW^{0.75}*\frac{\left(0.0384+0.04997*NEm{D_{d}}^{2}\right)}{NEmA}+0.2*Lact_{d}, \end{array}$$ (19)

else


$$\begin{array}{l} DMI_{d}=CSBW^{0.75}*\frac{\left(0.04631+0.04997*NEm{D_{d}}^{2}\right)}{NEmA}+0.2*Lact_{d}, \end{array}$$ (20)

where NEmD_*d*_ is the diet nutrient density (NEm/kg dry matter (DM)) on day *d* and NEmA = if NEmD_*d*_ ≥ 1, then NEmD_*d*_, else 0.95.

For weaned animals less than 2-yr-old DMI_*d*_ is determined by first calculating DMI as percent of body weight as follows (NASEM, 2016):


$$\begin{array}{l} DMI_{d}=\;\;\;DMI\;\;\;PCT_{d}*CSBW_{d}, \end{array}$$ (21)

where


$$\begin{array}{l} DMI\;\;\;PCT{\;\;\;}_{d}=\frac{1.2425+1.9218*NEmD_{d}\;\;\;--\;\;\;0.7259\;\;\;*NEm{D_{d}}^{2}}{100}. \end{array}$$ (22)

For each mean MW category, a maximum limit to DMI PCT is assigned according to Supplementary Table S1.8. Daily calf forage DMI is calculated from a slight modification to equation 25 in Tedeschi and Fox (2009). Equation 25 from Tedeschi and Fox (2009) tends to under-predict calf forage DMI, particularly at higher DMI levels, presumably at heavier calf weights and late in the lactation curve. In the present model, daily milk production (kg/d) replaced the static peak milk level variable in the original formulation. On average, Tedeschi and Fox (2009)equation 25 under-predicted calf forage DMI by 0.57 kg/d. Comparing the modified equation to the original equation at 6, 8, 10, and 12 kg/d PL over a 212-d nursing period with grazing nutrient density according to Supplementary Table S1.6, the modified equation predicted daily forage DMI an average of 0.65 kg/d greater than the original equation. Considering the 0.57 kg/d under-prediction of Tedeschi and Fox (2009), the modified equation increased calf forage DMI to better align with the actual calf intake data in Figure 5 of Tedeschi and Fox (2009).

Body weight gain (kg/d) and BCS (1 to 9) are calculated daily for each animal. Individual daily shrunk body gain (SBG_*jd*_) for animals ≤3-yr-old is calculated as follows (NASEM, 2016):


$$\begin{array}{l} SBG_{jd}=\frac{12.341*EB{W_{jd}}^{-0.6837}*NEg{I_{jd}}^{0.9116}+EBL_{jd}}{0.956}, \end{array}$$ (23)

where


$$\begin{array}{l} EBW_{jd}\;=\;0.891*EQSBW_{jd}, \end{array}$$ (24)

where


$$\begin{array}{l} EQSBW_{jd}\;=\frac{478}{MW_{j}}*CSBW_{jd}, \end{array}$$ (25)

$$\begin{array}{l} NEgI_{jd}=max(NEgD_{d}*\;\left(DMI_{jd}-NEmR\;\;\;DMI_{jd}\right),0), \end{array}$$ (26)

where NEgD_*d*_ is the net energy for gain (NEg) diet density (Mcal/kg DM) on day *d* and NEmR DMI_*jd*_ is the DMI required to meet NEmR.

Empty body weight loss (**EBL**) for animal *j* on day *d* is calculated by (NASEM, 2016):

If NEmI_*jd*_ < NEmR_*jd*_, then


$$\begin{array}{l} EBL_{jd}=\frac{NEmI_{jd}-NEmR_{jd}}{Mcal\;\;\;kg\;\;\;BCS\;\;\;loss}, \end{array}$$ (27)

else


$$\begin{array}{l} EBL_{jd}=0, \end{array}$$ (28)

where NEmI_*jd*_ is the NEm intake (Mcal) for animal *j* on day *d* and Mcal kg BCS loss is the Mcal required to lose 1 kg EBW at a given BCS according to Supplementary Table S1.9.

For animals ≤2-yr-old daily BCS is determined by calculating total body fat (BF; kg) as a percentage of SBW and then rounding to the nearest BCS according to Supplementary Table S1.9. Daily BF gain (BFG) is calculated by (NASEM, 2016):

If NEmI_*jd*_ > NEmR_*jd*_, then


$$\begin{array}{l} BFG_{d}=max(0.15*NEgI_{d}-0.0057*NEg{I_{d}}^{2}-0.162,\;\;\;0), \end{array}$$ (29)

else


$$\begin{array}{l} BFG_{d}=EBL_{d}. \end{array}$$ (30)

For cows 4-yr-old or greater, individual, daily shrunk body weight change is determined by the following:


$$\begin{array}{l} SBG_{jd}=\frac{EBG_{jd}+EBL_{jd}}{0.956}, \end{array}$$ (31)

where if NEmI_*jd*_ < NEmR_*jd*_, then


$$\begin{array}{l} EBL_{jd}=\frac{NEmI_{jd}-NEmR_{jd}}{Mcal\;\;\;kg\;\;\;BCS\;\;\;loss}, \end{array}$$ (32)

and


$$\begin{array}{l} EBG_{jd}=0, \end{array}$$ (33)

else


$$\begin{array}{l} EBL_{jd}=0 \end{array}$$ (34)

and


$$\begin{array}{l} EBG_{jd}=\frac{NEmI_{jd}-NEmR_{jd}}{Mcal\;\;\;kg\;\;\;BCS\;\;\;gain}, \end{array}$$ (35)

where Mcal kg BCS gain is the Mcal required to gain 1 kg EBW at a given BCS according to Supplementary Table S1.9.

For individual animals 4-yr-old or greater, daily BCS is determined by calculating CSBW as a percentage of mature shrunk body weight (MSBW; BCS 5) and rounding to the nearest BCS classification according to Supplementary Table S1.9. For 3-yr-old cows, BCS is calculated in both the manner for animals 2-yr and younger and the manner for 4-yr and older. The final daily BCS for 3-yr-olds is the maximum of the two methods. Decision rules are designed to maintain all post-weaning animals at BCS 5 or 6, although it is possible to fall outside that window under certain nutrient availability and intake conditions.

The Flint Hills bluestem based grazing season is parameterized to occur between May 1 and October 31 annually with forage nutrient density peaking in April to June at 1.48 Mcal NEm per kg dry matter (DM) and 0.9 Mcal NEg per kg DM (Kuhl et al., 1993; Supplementary Table S1.6). In July and August, forage Mcal NEm and NEg per kg DM are 1.1 and 0.54, respectively. September through March Mcal NEm and NEg per kg dry matter are 0.71 and 0.18, respectively (Kuhl et al., 1993; Supplementary Table S1.6). Total forage production per acre is estimated using a linear regression equation with cumulative precipitation for Manhattan, KS (HPRCC, 2019; Supplementary Table S1.4) between January and August and the percent of forage remaining at the end of the prior grazing season as input factors. The regression equation was estimated using Flint Hills grazing data from 1958 to 1965 (Launchbaugh and Owensby, 1978) paired with cumulative precipitation for Manhattan, KS for the corresponding years (HPRCC, 2019). Based on accumulated forage intake, forage production per acre, stocking rate, and a 25% forage waste parameter (Ogle and Brazee, 2009) animals are removed from grazing prior to November 1, if the percent of forage remaining falls below 40%. While grazing, if an animal, excluding nursing calves, is below BCS 5 with a negative energy balance she receives the 60% alfalfa, 40% corn supplement diet (1.63 Mcal NEm per kg dry DM, 1.02 Mcal NEg per kg DM) until her NEm and NEg requirements are met or at most 20% daily DMI.

Outside the grazing season, cows are fed a 73% alfalfa, 19% wheat straw, and 8% corn base diet with 1.2 Mcal NEm per kg DM and 0.64 Mcal NEg per kg DM. With the exception of nursing calves, if an animal has a BCS greater than 6, daily DMI is restricted to 70% of what is required to meet NEm requirements until the individual is BCS 6 or below. If a cow is below BCS 5 with a negative energy balance the supplement diet is added to her ration mix until NEm and NEg requirements are met, subject to a cost-minimizing linear programming model (MLPM) with DMI constraints. If there is no solution to the MLPM, the supplement diet replaces 40% of the base diet. The linear programming model is constructed as follows:

Minimize Objective Function:


$$\begin{array}{l} Ration\;\;\;Expense_{jd}=x_{jd}*BRP_{d}+y_{jd}*SRP_{d}, \end{array}$$ (36)

subject to constraints:


$$\begin{array}{l} x_{jd}*NEgBR+y_{jd}*NEgSR\geq NEgR_{jd}+NEgBR*\frac{NEmR_{jd}}{NEmBR}, \end{array}$$ cstr. (1)

$$\begin{array}{l} x_{jd}*NEgBR+y_{jd}*NEgSR\geq NEgR_{jd}+NEgSR*\frac{NEmR_{jd}}{NEmSR}, \end{array}$$ cstr. (2)

$$\begin{array}{l} x_{jd}*NEmBR+y_{jd}*NEmSR\geq NEmR_{jd}, \end{array}$$ cstr. (3)

$$\begin{array}{l} x_{jd}*\frac{1}{DMI\;\;\;PCT_{jd}}+y_{jd}*\frac{1}{DMI\;\;\;PCT_{jd}}\geq CSBW_{jd}, \end{array}$$ cstr. (4)

$$\begin{array}{l} x_{jd}+y_{jd}\leq DMI_{jd}, \end{array}$$ cstr. (5)

$$\begin{array}{l} x_{jd}\leq Available\;\;\;BR_{jd}, \end{array}$$ cstr. (6)

$$\begin{array}{l} x_{jd}\geq 0, \end{array}$$ cstr. (7)

$$\begin{array}{l} y_{d}\geq 0. \end{array}$$ cstr. (8)

Constraints 1 and 2 ensure that NEg in the final diet mix for animal *j* on day *d* meets or exceeds the NEg requirement for animal *j* on day *d*. Constraint 3 ensures that NEm in the final diet mix for animal *j* on day *d* meets or exceeds the NEm requirement for animal *j* on day *d*. DMI as a percent of shrunk body weight is controlled by constraint 4. Constraint 5 ensures that DMI (kg) does not exceed that previously calculated according to NASEM (2016) equations and model rules as previously described. Constraint 6 holds base ration intake equal to or less than the maximum available allocation for animal *j* on day *d*. Non-negativity is controlled by constraints 7 and 8. See Tables 1 and 2 for MLPM variable description.

**Table 1.** MLPM decision variable descriptions

Variable	Description
*x*_*jd*_	Animal *j* base diet intake on day *d*, kg DM
*y*_*jd*_	Animal *j* supplement diet intake on day *d*, kg DM

**Table 2.** MLPM parameter variable descriptions

Variable	Description
Ration Expense_*jd*_	Animal *j* ration expense, USD, on day *d*
BRP_*d*_	Base diet price on day *d*, USD/kg
SRP_*d*_	Supplement diet price on day *d*, USD/kg
NEgBR	Base diet NEg density, Mcal/kg DM
NEgSR	Supplement diet NEg density, Mcal/kg DM
NEgR_*jd*_	Animal *j* required NEg on day *d*, Mcal
NEmR_*jd*_	Animal *j* required NEm on day *d*, Mcal
DMI PCT_*jd*_	Animal *j* DMI as percent of *CSBW*_*jd*_ on day *d*
CSBW_*jd*_	Animal *j* CSBW on day *d*, kg
Available BR_*jd*_	Maximum base diet available, kg, on day *d* for animal *j* based on animal type according to Supplementary Table S1.10

Calf nutrition calculations and rules are similar to those of mature animals, with the addition of milk in the diet per the dam’s daily production. Mcal NEm and NEg per kg liquid milk is set to 0.55 and 0.33, respectively (Parkins et al., 1977). The model assumes that calves consume only milk through 50 d postpartum. Calves are not offered creep feed, but will be fed the cow supplement diet as needed if pulled off grass before weaning. Daily calf DMI is calculated from Tedeschi and Fox (2009), as previously described. Pertinent to the Tedeschi and Fox (2009) equation, forage Mcal digestible energy (DE) per kg is 2.86 for April-June, 2.12 for July and August, and 1.89 for September through March. Fed diet DE is 3.08 Mcal/kg for all months (Supplementary Table S1.6).

### Reproduction

Age at first estrus for each individual replacement heifer is the maximum of a randomly drawn value from a normal distribution with mean = 300 and SD = 20 (Diskin and Kenny, 2014) or the day a heifer reaches 40% of her mature weight (Davis and Wettemann, 2009).

Each cow’s postpartum interval (PPI; d between calving and first postpartum estrus) is drawn from a pert distribution according to parity, BCS, and dystocia (Supplementary Table S1.11; Bellows et al., 1988; Berardinelli et al., 2005; Ciccioli et al., 2003; Cushman et al., 2007; Doornbos et al., 1984; Endecott et al., 2007; Graham, 1982; Houghton et al., 1990; Lents et al., 2008; Rutter and Randel, 1984). If a cow experiences dystocia, the subsequent increase in her PPI is drawn from a truncated random normal distribution with mean = 10 d, SD = 3 d, and lower bound = 0 d (Bellows et al., 1988; Doornbos et al., 1984).

The mean herd-wide dystocia risk for all multiparous cows and primiparous cows with calf BW < 90 pounds (40.82 kg) is 0.05 (SD = 0.01, lower bound = 0; McDermott et al. 1992; USDA, 2008). The mean herd wide dystocia risk for primiparous cows with calf BW ≥ 90 pounds is 0.08 (SD = 0.01, lower bound = 0).

Following the first estrus for a given production year, each individual’s estrous cycle is simulated with d between estrus drawn from a truncated normal distribution with mean = 21, SD = 0.75, upper bound = 24, and lower bound = 18. If a female is not pregnant and an estrus day occurs during the breeding season she has an opportunity to become pregnant (Supplementary Table S1.11). After becoming pregnant there is daily abortion risk (Supplementary Table S1.11). If a female aborts before the end of the breeding season, she has the opportunity to establish one more pregnancy. Preliminary gestation length for each pregnant female is randomly drawn from a normal distribution (mean = 285 d, SD = 5), before being adjusted through Cholesky decomposition methods to match the +0.30 genetic correlation between calf BW and gestation length (Gregory et al., 1995; Hofert, 2013).

### Health

Calf morbidity and calf mortality prior to weaning are binary outcomes determined daily. Within the model, a calf may experience morbidity once, at most. The daily morbidity probability for each calf within a simulation iteration is drawn from truncated random normal distributions based on calf age and dystocia occurrence (Supplementary Table S1.13). The daily probability of mortality is drawn from a truncated random normal distribution. Eight different distributions represent potential combinations of age, dystocia occurrence, and mortality occurrence (Supplementary Table S1.13). For both morbidity and mortality, calf age is separated into neonatal (≤3 d old) and post-neonatal to weaning (>3 d old to weaning). The same daily probability for morbidity and mortality applies to each calf within the same category (age, dystocia occurrence, prior morbidity) for all years within a single simulation iteration.

Mortality is a daily, binary outcome for both post-weaning replacement heifers (before establishing first pregnancy) and females in the breeding herd. The two age categories have different truncated random normal distributions from which daily mortality risk is drawn at the start of each simulation iteration (Supplementary Table S1.13). For a given iteration, the same daily mortality risk applies to each female.

### Validation Procedures

Three different validation procedures were applied to simulation output for production years 2000 through 2018:

1) Expert panel review,
2) Descriptive statistics comparing model output to historical cow–calf production data, and
3) Exploratory factor analysis on a combined data set of model output and actual historical cow–calf production data.

#### Expert panel review

The model was simulated 100 iterations per MW and PL combination scenario according to the previously described parameters and design. Graphical output for 17 different simulation outcome variables by MW and PL combination were presented to a six-person panel with combined expertise spanning veterinary medicine, animal breeding and genetics, ruminant nutrition, agricultural economics, and beef production modeling. All panelists were faculty at Kansas State University. Three of the six panelists are also co-authors of this work who had contributed to the theory behind model design, but were naïve to model outcomes prior to panel review. Table 3 provides further expert panel details. Variable distributions were aggregated across production years 2000 through 2018 and median values were displayed by cow type and production year. Variables presented ranged from growth and reproductive traits to nutrition and herd demographics. Each output variable was reviewed and discussed in absolute value terms and relative to other scenarios.

**Table 3.** Expert panel qualifications and experience

Expert panel reviewer	Qualifications	Years of professional experience
1	DVM, PhD (Animal Sciences and Industry)	>30
2	PhD (Animal Breeding)	>25
3	PhD (Agricultural Economics)	>15
4	DVM, MS	>20
5	DVM, PhD (Epidemiology)	>15
6	PhD (Ruminant Nutrition)	>10

#### Descriptive statistics: model output vs. actual production data

There are very few multiyear, multiherd, commercial cow–calf datasets that track multiple variables for individual animals applying the standardization of beef cow–calf standardized performance analysis (**SPA**) methodology. North Dakota State University’s CHAPS program (CHAPS, 2020) meets such qualifications and was made available to the authors. While environmental and grazing conditions are specific to the Kansas Flint Hills, the model goal of meeting breeding female nutrient requirements by maintaining a BCS score of 5 or 6 through supplementation implies that stocking rate and grazing nutrient availability in the Flint Hills should not impact animal performance. Rather, the impact will be manifest in feed costs through fed ration requirements. The exception to this rule is the nutritional content of Flint Hills grass for nursing calves, as they are not allowed supplementation until after weaning (i.e., no creep feeding).

Given the previously described prevalence of the Angus breed in the U.S. commercial cow herd, the use of U.S average Angus genetic parameters, and the model goal of meeting animals’ nutritional needs, it is reasonable to expect modeled output to fall within the distribution of CHAPS data.

#### Individual data

CHAPS individual calf data from the years 2000 through 2017 was paired with individual cow data from the same data set by matching herd, year, and cow identification number. Variables considered were actual calf birth weight (ABW), pre-weaning ADG, calf weaning age (WAGE), calf 205 d adjusted weaning weight (ADJ WW), cow age (CAGE), and cow weight at calf weaning (MWW). All weight variables were converted to kg at 2.205 pounds equals 1 kg. MWW was classified by rounding to the nearest simulated scenario MW class (e.g., 560 kg MWW rounds to 544 kg MW class). After classifying to MW, MWW was no longer considered. The model assumes cows reach maturity at 4-yr-old. Thus, all CHAPS records with CAGE less than 4 were removed to prevent confounding MWW with maturity. Although BCS at weaning is a potential variable, few cows in the paired data set had a BCS record. Therefore, BCS was not considered. The model simulation was parameterized to describe U.S. average Angus BW and WW genetics; however, implementing an explicit Angus breed identification requirement to the paired data in addition to the other described data requirements yielded only 235 records. After all qualifications, the data set consisted of 5,025 unique records of ABW, WAGE, pre-weaning ADG, ADJ WW, CAGE, and MW from 1,642 individual cows and 6 herds. All records were from North Dakota. Table 4 reports the percent of total records in each MW category for the final data set (IND CHAPS).

**Table 4.** Percent of total records in each MW category for IND CHAPS data set consisting of 5,025 unique records from 1,642 cows and 6 herds

MW category, kg	Percent of total records, %
454	0.7
499	2.9
544	8.3
590	17.5
635	19.3
680	20.5
726	15.9
771	14.8

With variables matching IND CHAPS, 5,025 total records were randomly sampled (IND MOD) from a combined dataset of all cow types and production years 2000 to 2017, to create a balanced data set for EFA. No requirements were applied for the percent of sample in MW category (Table 5) or number of unique cows. Box-plots identifying the median, 25th percentile, and 75th percentile for each variable by data source (IND CHAPS or IND MOD) and MW category were generated and evaluated.

**Table 5.** Percent of total records in each MW category for the model sample data set consisting of 5,025 unique records

MW category, kg	Percent of total records, %
454	13.1
499	12.3
544	12.7
590	13.1
635	11.6
680	10.8
726	13.1
771	13.4

#### Herd data

Yearly herd-level data was also provided by the CHAPS program (CHAPS, 2020). Using herd data from 2000 to 2017, selected yearly records were required to expose 75 or more females for breeding and have complete records for mean actual calf birth weight (MBRTWT), for mean calf weaning age (MAGE), mean pre-weaning ADG (MADG), mean cow age (MCAGE), percent pregnant per cow exposed (PREGPERC), percent pregnancy loss per cow exposed (PREGLP), and percent calf mortality per calf born (CALFL2). A total of 422 unique yearly records from 39 herds met the specifications (HERD CHAPS). North Dakota, South Dakota, Iowa, and Kansas were represented by 79.1%, 11.6%, 5.7%, and 3.6% of HERD CHAPS records, respectively. Data scarcity for mean cow weight and mean cow BCS in the CHAPS dataset prohibited their inclusion in HERD CHAPS.

To generate a comparable sample from model simulated output, yearly herd-level data for production years 2000 through 2017 was selected from one iteration each of the 32 simulated MW and PL combination scenarios. All 18 yr from each of the 32 scenarios were pooled together. To balance the model output with the CHAPs data in subsequent EFA, 422 yearly records were randomly selected (HERD MOD). Box-plots identifying the median, 25th percentile, and 75th percentile for each variable by MW-PL combination were generated. HERD CHAPS records remained aggregated as cow type was not identified.

### Exploratory Factor Analysis: Model Output vs. Actual Production Data

#### Individual data

IND CHAPS and IND MOD were combined to form a data set of 10,050 ABW, WAGE, ADJ WW, CAGE, and MW records. Pre-weaning ADG was omitted as it is inherently included in ADJ WW. The psych package (Revelle, 2019) was applied in R statistical software (R Core Team, 2019) to perform EFA. Including both modeled and actual data in the same EFA assumes a naïve expectation by applying EFA without a preconceived bias regarding which population a vector of variables belongs to. Therefore, any distinction between IND CHAPS and IND MOD is determined objectively through EFA. Principal axes factoring (PAF) was used to extract two latent factors from the combined individual data. PAF does not rely on multivariate normality assumptions (Osborne, 2014). The maximum PAF iterations parameter was set to 100; however, convergence occurred before the 100th iteration. The number of factors to extract, two, was determined through parallel analysis (Osborne, 2014). Factor loadings, which are the standardized regression coefficients of observed variables regressed on the underlying, extracted latent factors, were rotated by “oblimin” rotation (Osborne, 2014; Revelle, 2019). Factor scores, each individual’s numerical factor values, were estimated using the Thurstone method (Grice, 2001; Thurstone, 1935).

#### Herd data

HERD CHAPS and HERD MOD were combined to form a data set with 844 records. PAF was applied to extract three latent factors. All other EFA methods match those already described.

## RESULTS AND DISCUSSION

### Expert Panel Review

The expert panel had no objections to model output, agreeing that both the absolute and comparative output seemed reasonable given their experiences and expectations.

### Descriptive Statistics: Model Output vs. Actual Production Data

#### Individual data

**Figure 1.**
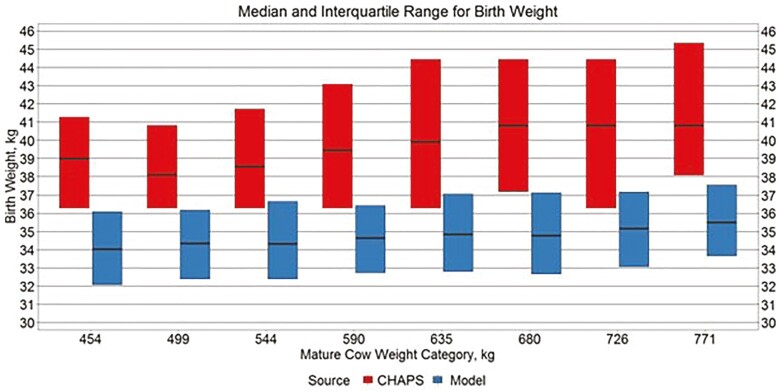
Median and interquartile range for birth weight. Boxplots representing the median, 25th percentile, and 75th percentile for individual calf birth weight from IND CHAPS and IND MOD.

**Figure 2.**
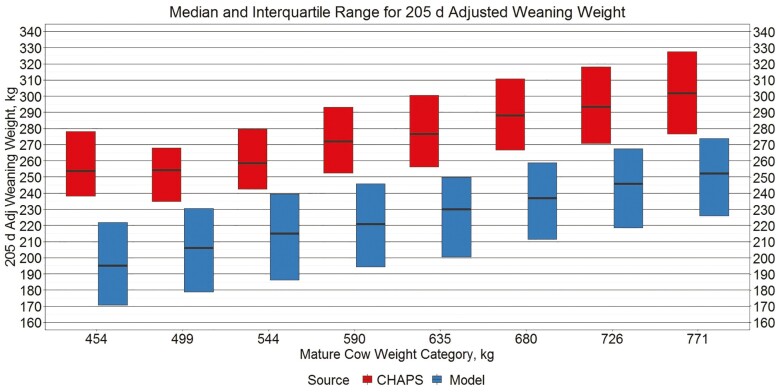
Median and interquartile range for 205 d adjusted weaning weight. Boxplots representing the median, 25th percentile, and 75th percentile for individual calf 205 d adjusted weaning weight from IND CHAPS and IND MOD.

Median ABW across all MW categories was 39.9 kg and 34.8 kg for IND CHAPS and IND MOD, respectively. Median ADJ WW across all MW categories was 281.6 and 225.3 kg for IND CHAPS and IND MOD, respectively. The interquartile boxplots displayed in Figures 1 and 2 show that compared to the six CHAPS herds contributing to IND CHAPS, model output for ABW and ADJ WW was lower across all MW categories. Subtracting the median IND CHAPS pre-weaning average daily gain from the median IND MOD pre-weaning average daily gain for each of the eight respective mature cow weight genetics categories, and then calculating the median of the eight values, the median difference was −0.21 kg/d. Performing the same calculation for birth weight and adjusted 205 d weaning weight, IND MOD was 4.9 and 48.6 kg lighter than IND CHAPS, respectively. Such a difference could be attributed to a number of factors including differences in growth genetics and nutrient availability. One quarter of the modeled scenarios include PL genetics at the low 6.8 kg/d level. The presence or absence of creep feed was not identified in CHAPS data. Although it may exist, an adequate daily growth model based on NEm and NEg intake for nursing calves was not found in the literature. Thus, growth equations for heavier calves from NASEM (2016) were extrapolated to lighter weights. Furthermore, most growth equations are based on decades-old research that may not describe the current beef cattle population as well as the population upon which they were derived.

Nonetheless, model output for ABW and ADJ WW still falls in the range of values found in the industry. Simultaneously, greater values for ABW and ADJ WW by MW in IND CHAPS compared to the same measures in IND MOD is consistent with the 0.29 genetic correlation between birth weight and weaning weight in the modern Angus population (AAA, 2019c). The rate of change in ABW and ADJ WW across different MW categories also appears similar between data sources suggesting that model comparisons between cow types could be informative.

**Figure 3.**
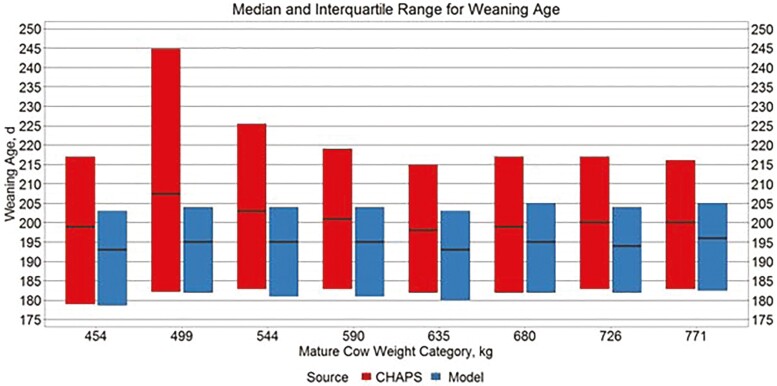
Median and interquartile range for weaning age. Boxplots representing the median, 25th percentile, and 75th percentile for individual calf weaning age from IND CHAPS and IND MOD.

**Figure 4.**
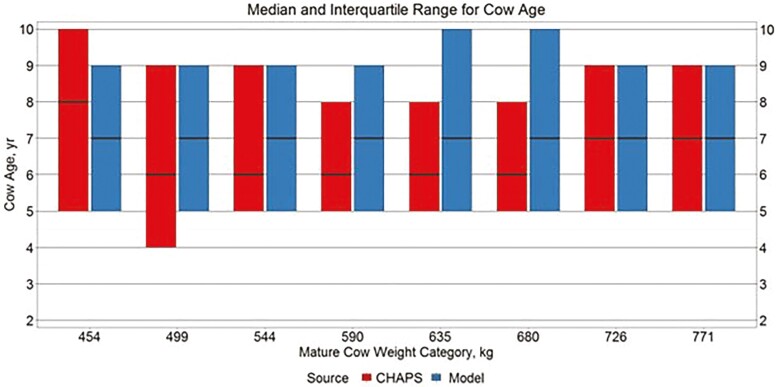
Median and interquartile range for cow age. Boxplots representing the median, 25th percentile, and 75th percentile for individual cow age from IND CHAPS and IND MOD.

Median WAGE and CAGE (Figures 3 and 4) appeared relatively similar between data sources, although WAGE showed a wider interquartile range across all MW categories in IND CHAPS compared to IND MOD. This finding is reasonable considering the only difference in modeled scenarios is MW-PL combination, while herd-to-herd and year-to-year management decisions likely vary within CHAPS data.

#### Herd data

**Figure 5.**
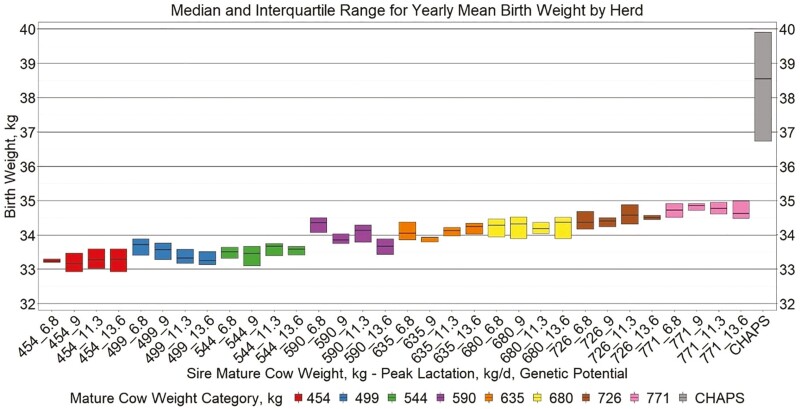
Median and interquartile range for yearly mean birth weight by herd. Boxplots representing the median, 25th percentile, and 75th percentile for yearly mean calf birth weight by cow MW and PL category combination from Herd MOD and HERD CHAPS. MW and PL category could not be identified for HERD CHAPS.

**Figure 6.**
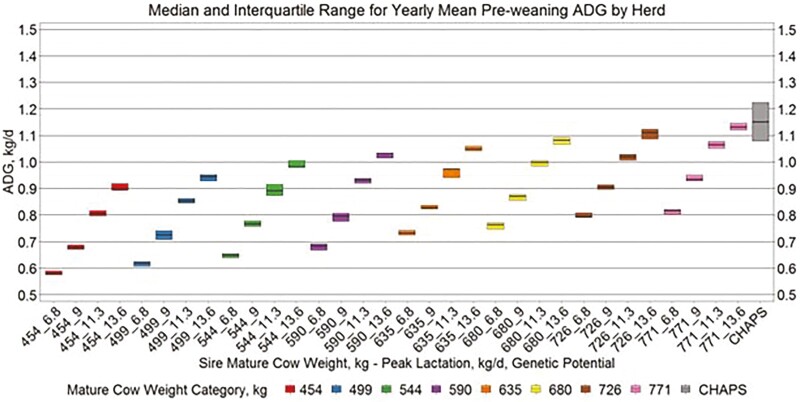
Median and interquartile range for yearly mean pre-weaning ADG by herd. Boxplots representing the median, 25th percentile, and 75th percentile for yearly mean pre-weaning ADG by cow MW and PL category combination from HERD MOD and HERD CHAPS. MW and PL category could not be identified for HERD CHAPS.

**Figure 7.**
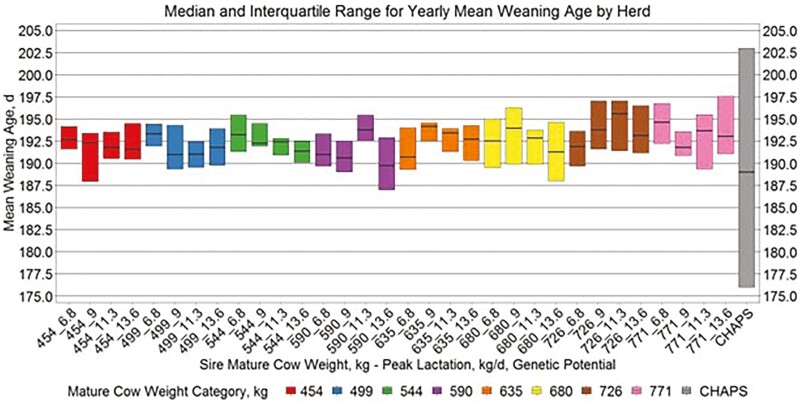
Median and interquartile range for yearly mean weaning age by herd. Boxplots representing the median, 25th percentile, and 75th percentile for yearly mean weaning age by cow MW and PL category combination from HERD MOD and HERD CHAPS. MW and PL category could not be identified for HERD CHAPS.

**Figure 8.**
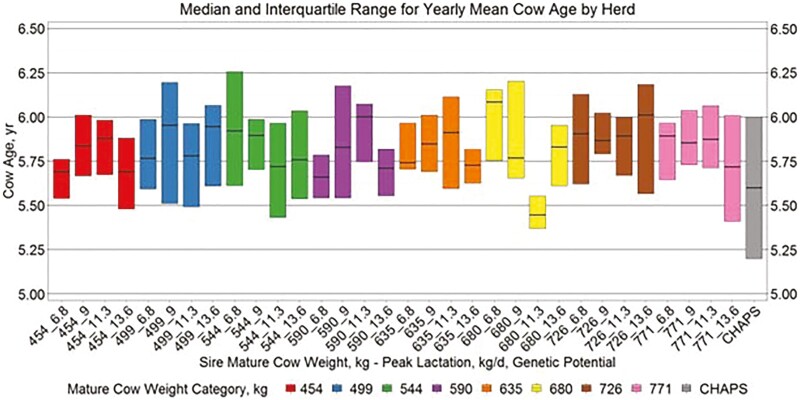
Median and interquartile range for yearly mean cow age by herd. Boxplots representing the median, 25th percentile, and 75th percentile for yearly mean cow age by cow MW and PL category combination from HERD MOD and HERD CHAPS. MW and PL category could not be identified for HERD CHAPS.

**Figure 9.**
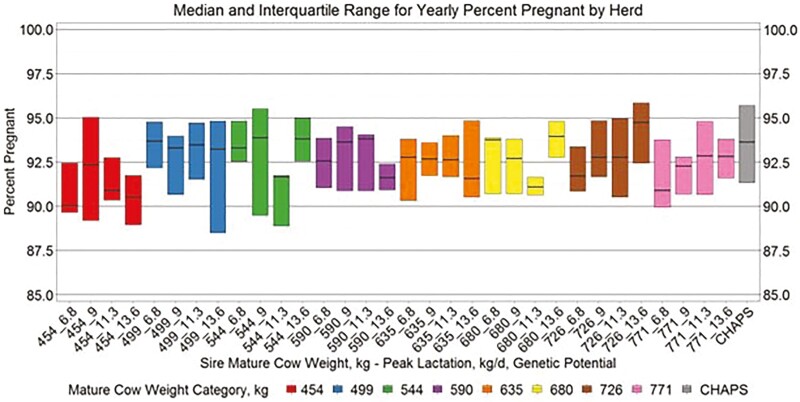
Median and interquartile range for yearly percent pregnant by herd. Boxplots representing the median, 25th percentile, and 75th percentile for percent pregnant per cow exposed by cow MW and PL category combination from HERD MOD and HERD CHAPS. MW and PL category could not be identified for HERD CHAPS.

**Figure 10.**
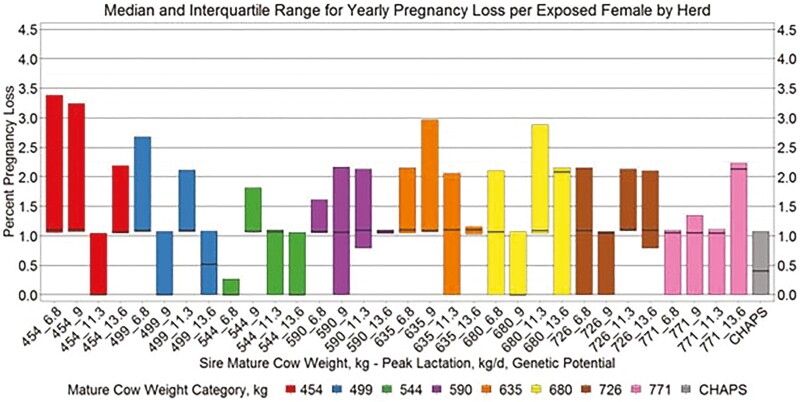
Median and interquartile range for yearly pregnancy loss per exposed female by herd. Boxplots representing the median, 25th percentile, and 75th percentile for percent pregnancy loss per cow exposed by cow MW and PL category combination from HERD MOD and HERD CHAPS. MW and PL category could not be identified for HERD CHAPS.

**Figure 11.**
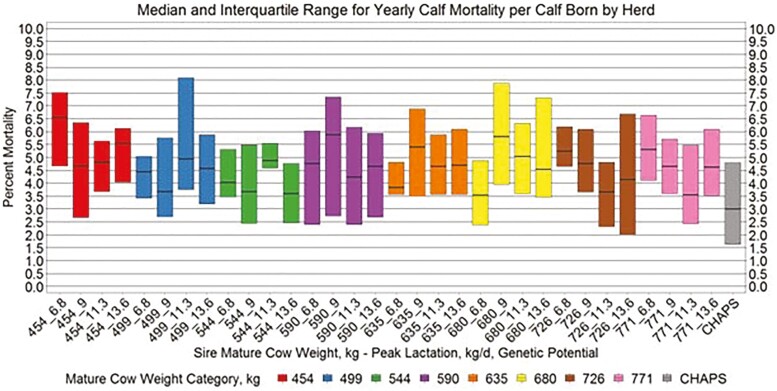
Median and interquartile range for yearly calf mortality per calf born by herd. Boxplots representing the median, 25th percentile, and 75th percentile for percent calf mortality per calf born by cow MW and PL category combination from HERD MOD and HERD CHAPS. MW and PL category could not be identified for HERD CHAPS.

Yearly birth weight, growth, and age variables by herd (Figures 5–8) suggest similar conclusions to those discussed at the individual animal level. Median MAGE across all MW categories was 189 and 192.4 d for HERD CHAPS and HERD MOD, respectively. Median MCAGE across all MW categories was 5.6 yr and 5.8 yr for IND CHAPS and IND MOD, respectively. Wider interquartile ranges in Figures 5–7 for HERD CHAPS are reasonable considering the data are not identified by cow type and the likely management variation across the 39 herds contributing to HERD CHAPS. Reproduction and survivability traits appear comparable between data sources (Figures 9–11) with a slight advantage to the CHAPS herds. Median PREGPERC across all MW categories was 93.7% and 92.7% for HERD CHAPS and HERD MOD, respectively. Across all MW categories, the median PREGLP and CALFL2 were 0.4% and 3% for HERD CHAPS, and 1.1% and 4.7% for HERD MOD. With the model designed and parameterized to describe U.S. industry averages, it is not surprising that herds submitting CHAPS data display an advantage in several traits, if stringent record keeping is associated with progressive, diligent management.

### Exploratory Factor Analysis: Model Output vs. Actual Production Data

#### Individual data

**Figure 12.**
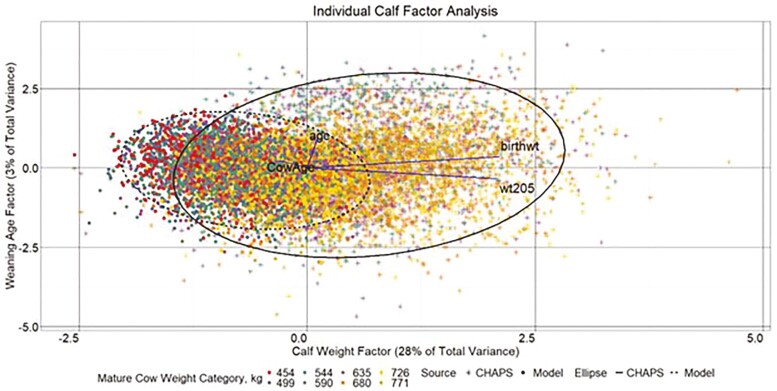
Individual calf factor analysis. Individual calf factor score plot. birthwt, actual calf birth weight (ABW); wt205, calf 205 d adjusted weaning weight (ADJ WW); age, calf weaning age (WAGE); and CowAge, cow age (CAGE). Line vectors represent variables’ direction and magnitude of influence based on their factor loadings, which are rescaled by multiplying each variable factor loading by 2.85. Data ellipses represent the two SD factor score coordinate boundary.

About 31% of total variance in the combined individual data set can be attributed to the two latent factors extracted by EFA. The variable factor loadings plotted in Figure 12 (line vectors) suggest a primary underlying factor influencing ABW and ADJ WW. Another factor appears to influence weaning age. Factor scores for all 10,050 records are plotted in Figure 12. Multivariate normal data ellipses spanning two SD are drawn around each data source. Similar to analysis through descriptive statistics, EFA suggests increased ABW and ADJ WW for calves from the CHAPS data with IND MOD data from heavier MW categories aligning more closely with IND CHAPS. Similar age traits match the descriptive statistics as well. The consistent generalities between descriptive statistics and EFA are reassuring.

#### Herd data

**Figure 13.**
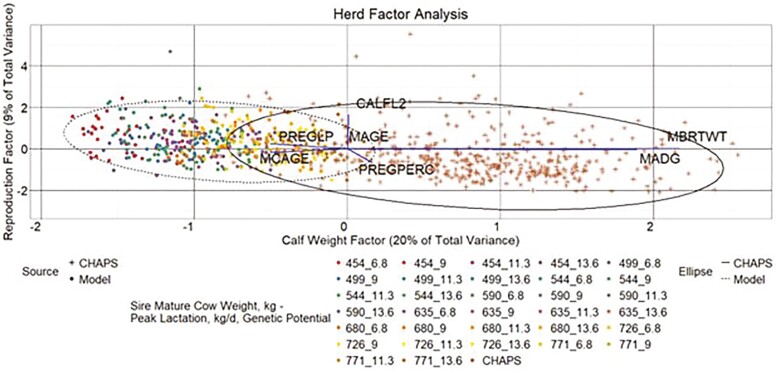
Herd factor analysis. Yearly herd data factor score plot reproduction factor by calf weight factor. MBRTWT, mean actual calf birth weight; MAGE, mean calf weaning age; MADG, mean pre-weaning ADG; MCAGE, mean cow age; PREGPERC, percent pregnant per cow exposed; PREGLP, percent pregnancy loss per cow exposed; and CALFL2, percent calf mortality per calf born. Line vectors represent variables’ direction and magnitude of influence based on their factor loadings, which are rescaled by multiplying each variable factor loading by 2.5. Data ellipses represent the two SD factor score coordinate boundary.

**Figure 14.**
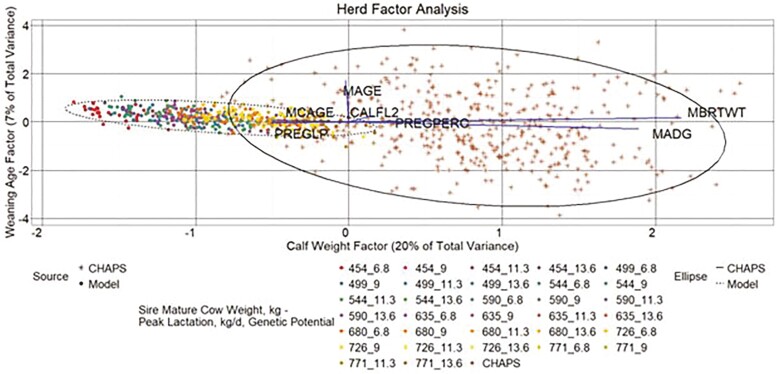
Herd factor analysis. Yearly herd data factor score plot weaning age factor by calf weight factor. MBRTWT, mean actual calf birth weight; MAGE, mean calf weaning age; MADG, mean pre-weaning ADG; MCAGE, mean cow age; PREGPERC, percent pregnant per cow exposed; PREGLP, percent pregnancy loss per cow exposed; and CALFL2, percent calf mortality per calf born. Line vectors represent variables’ direction and magnitude of influence based on their factor loadings, which are rescaled by multiplying each variable factor loading by 2.5. Data ellipses represent the two SD factor score coordinate boundary.

**Figure 15.**
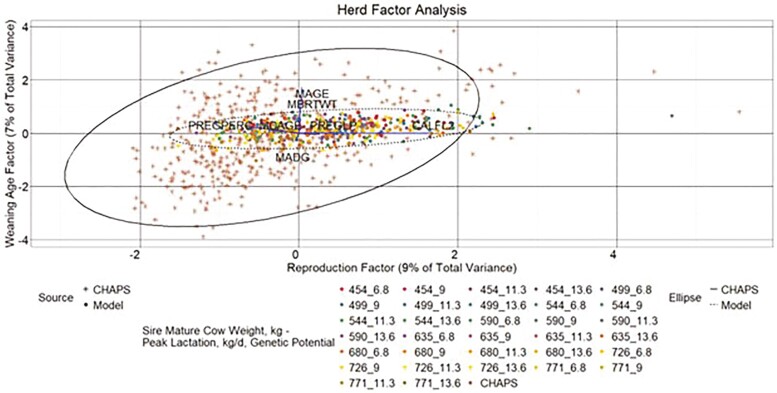
Herd factor analysis. Yearly herd data score plot weaning age factor by reproduction factor. MBRTWT, mean actual calf birth weight; MAGE, mean calf weaning age; MADG, mean pre-weaning ADG; MCAGE, mean cow age; PREGPERC, percent pregnant per cow exposed; PREGLP, percent pregnancy loss per cow exposed; and CALFL2, percent calf mortality per calf born. Line vectors represent variables’ direction and magnitude of influence based on their factor loadings, which are rescaled by multiplying each factor loading by 2.5. Data ellipses represent the two SD factor score coordinate boundary.

A three**-**factor model described 36% of the total variance in the combined herd-level data (Figures 13–15). A common factor that seemingly influences calf weight accounted for 20% of total variance. Factors that appear to describe reproductive and age traits accounted for 9% and 7% of total variance, respectively. As with the individual data EFA, CHAPS herds tended to have higher calf weight factor scores than modeled herds, while the calf weight factor scores from heavier MW categories in HERD MOD more closely aligned with HERD CHAPS than the lighter MW categories. Aligned with the descriptive statistics, HERD CHAPS and HERD MOD reproductive factor scores, driven largely by CALFL2 and PREGPERC, were more comparable than the calf weight scores. Also consistent with the descriptive statistics, the weaning age score for HERD MOD was in a much tighter range than HERD CHAPS.

#### General

The stochastic, individual based systems simulation model described offers a unique opportunity to simultaneously account for genetics, nutrition, reproduction, growth, and health in beef cow–calf production settings. The model’s stochastic elements consider the biological variation inherent to beef production which has tremendous advantages compared to deterministic techniques that ignore probabilistic risk and uncertainty. The systems design accounts for component interactions, as well as beef production’s time delays and its complex, prolonged feedback structure. These capabilities make the model ideal for decision analysis through the assessment of multiple metrics simultaneously, over various time horizons. In addition, the model can be expanded to include stocker, backgrounder, and finishing phases of the beef production system.

The primary difference between IND MOD and IND CHAPS was smaller ABW and smaller ADJ WW in the modeled output. In the herd data, the primary difference was smaller MBRTWT and MADG. By assuming purebred Angus genetics, the model does not include any heterosis effects, which are likely present in the CHAPS herds. Furthermore, the data used to estimate the model’s base birth weight equation and the Angus birth weight genetics incorporated into the model are from seedstock operations that likely place greater selection pressure on birth weight than the commercial operations represented in CHAPS. Whereas birth weight is not an economically relevant trait in commercial operations, assuming no dystocia, many seedstock breeders would contend that actual birth weight and birth weight EPD has a large impact on the market value of their genetics. While ABW in IND MOD tends to be about 5 kg smaller than in IND CHAPS, for each respective MW category (Figure 1), ABW in IND MOD does align relatively well with BIF male and female baseline birth weights of 34.0 kg and 31.2 kg, respectively (BIF, 2010). In weaned, pen-fed growing cattle, Fox et al. (2004) used three different models accounting for various intake, nutrient density, and digestibility parameters to predict ADG when either metabolizable energy or metabolizable protein was the first limiting factor in high forage diets. The prediction error of the modeled means minus the observed means ranged from −0.01 to 0.14 kg/d (Fox et al., 2004). Upon subtracting IND CHAPS MADG from IND MOD MADG for each respective MW category and then averaging the eight values, the average mean prediction error was −0.23 kg/d. The present research compares modeled vs. actual preweaning data rather than postweaning data. Furthermore, the models presented in Fox et al. (2004) are fit to known animal, nutrition, and environmental variables, while very little animal and no nutrition or environmental specifics are known in the CHAPS data. Regarding the CPCPM beef production model, Shafer et al. (2005) cited the inability to apply identical treatment effects to entire production systems over long time periods and limited whole system production data as reasons to forego formal model validation against observed data.

The lack of discussion on the interaction between calf preweaning nutrition and calf preweaning growth in NASEM (2016) illustrates a shortfall in research and understanding. The nature of beef cow–calf production and the challenge in determining preweaning calf milk and feedstuff intake exacerbates the difficulty. In the present model, the equation used to determine preweaning calf DMI was estimated using Holstein steers (Tedeschi and Fox, 2009), while the equation predicting growth based on nutrient intake was estimated postweaning (NASEM, 2016). As research regarding preweaning beef calf nutrition and growth advances, there may be opportunity to improve upon the present model.

At best, a model usefully simplifies reality’s complexity. The present model represents a mathematical interpretation of the current understanding regarding cow–calf production biology (i.e., a mathematical literature review). As such, any question a reader may have regarding model output validity or author assumptions may point to potential gaps in cow–calf production research, although opportunities for model improvements should not be ignored. Although some relative differences between modeled output and validation data appear, the absolute values of the modeled output still fall well within the realm of feasibility. Furthermore, the relative trends across MW and PL categories fit expectations, supporting the model’s usefulness as a tool for comparisons.

By stochastically accounting for the simultaneous interaction of nutrition, genetic, reproduction, and health variables at the individual level within a whole herd production system, the present model provides a unique decision-making tool when considering cow–calf production strategies. The model’s flexible design also allows for additional functions to be incorporated. With the addition of economic values, the model can concurrently assess biological and economic efficiencies over any user-defined time frame (Lancaster and Larson, 2022). It is also feasible to integrate land use, water use, and methane emissions into the model to evaluate individual and cow–calf system-wide resource and emissions efficiency based on genetic traits and management schemes (Lakamp et al., 2022). The ability to assess alternate genetic and management strategies with associated biological uncertainties in a system-wide manner should prove a valuable feature as cattle production faces continuous pressure to become more resource efficient in an everchanging physical and economic environment.

## Supplementary Material

txac155_suppl_Supplementary_Appendix

txac155_suppl_Supplementary_Figure_S1
