# Supplementary material for: Stochastic, individual animal systems simulation model of beef cow–calf production: development and validation
Source: Transl Anim Sci. 2022 Dec 3;7(1):txac155. doi: 10.1093/tas/txac155 (PMC9930734; doi:10.1093/tas/txac155)
Supplement: txac155_suppl_Supplementary_Appendix [file txac155_suppl_supplementary_appendix.docx]

**Appendix 1: Model Parameters**

**Table A1.1.** Initialization parameters.

| Model Parameter | Distribution Type | Distribution Parameters | Reference | Notes |
| --- | --- | --- | --- | --- |
| Iterations | Deterministic | 100 | User-defined | The number of iterations the user wishes to run an *i* production year simulation |
| Initial breeding herd size at calving | Deterministic | 100 | User-defined |  |
| Breeding herd size goal | Deterministic | 100 | User-defined | The breeding herd size the user wishes to achieve. |
| Years to herd size | Deterministic | 1 | User-defined | The number of years the user wishes to pass in achieving the breeding herd size goal. |
| Initial heifer replacement rate | Deterministic | 0.125 | User-defined (default from Wittum et al.(1994), USDA (2010), Cushman et al. (2013), Ringwall (2014), and expert opinion) |  |
| Breeding season start | Deterministic | May 1st | User-defined |  |
| Breeding season end | Deterministic | July 3 | User-defined |  |

**Table A1.2.** Four-year rolling average Angus genetic trend for birth weight and weaning weight from 1992 to 2018.

| Year | Angus Birth Weight EBV: Four Year Rolling Average, pounds | Angus Weaning Weight EBV: Four Year Rolling Average, pounds |
| --- | --- | --- |
| 1992 | 3.65 | 24.0 |
| 1993 | 3.80 | 27.0 |
| 1994 | 3.85 | 30.0 |
| 1995 | 3.80 | 32.5 |
| 1996 | 3.75 | 35.5 |
| 1997 | 3.75 | 38.0 |
| 1998 | 3.75 | 41.0 |
| 1999 | 3.80 | 44.5 |
| 2000 | 3.80 | 47.5 |
| 2001 | 3.80 | 51.0 |
| 2002 | 3.80 | 54.0 |
| 2003 | 3.80 | 57.0 |
| 2004 | 3.75 | 60.0 |
| 2005 | 3.7 | 62.5 |
| 2006 | 3.65 | 65.5 |
| 2007 | 3.55 | 68.5 |
| 2008 | 3.50 | 72.0 |
| 2009 | 3.40 | 75.5 |
| 2010 | 3.30 | 79.0 |
| 2011 | 3.20 | 82.0 |
| 2012 | 3.10 | 85.0 |
| 2013 | 3.00 | 88.0 |
| 2014 | 2.90 | 91.0 |
| 2015 | 2.85 | 94.5 |
| 2016 | 2.75 | 98.0 |
| 2017 | 2.70 | 102.0 |
| 2018 | 2.60 | 106.0 |

Adapted from AAA (2019b)

**Table A1.3.** Genetic correlations.

| Traits | Genetic Correlation | Reference |
| --- | --- | --- |
| Weaning Weight: Mature Cow Weight | 0.44 | AAA (2019c) |
| Birth Weight: Weaning Weight | 0.29 | AAA (2019c) |
| Gestation: Birth Weight | 0.30 | Gregory et al. (1995) |
| Milk Production: Mature Cow Weight | 0.14 | Morris and Wilton (1976) |

**Table A1.4.** Manhattan, KS January through August Cumulative Precipitation.

| Year | Jan- Aug Cumulative Precipitation, in |
| --- | --- |
| 1995 | 34.83 |
| 1996 | 23.61 |
| 1997 | 20.17 |
| 1998 | 24.15 |
| 1999 | 30.50 |
| 2000 | 15.43 |
| 2001 | 30.82 |
| 2002 | 18.43 |
| 2003 | 25.56 |
| 2004 | 32.54 |
| 2005 | 26.45 |
| 2006 | 26.2 |
| 2007 | 34.62 |
| 2008 | 33.07 |
| 2009 | 29.46 |
| 2010 | 26.70 |
| 2011 | 21.33 |
| 2012 | 17.46 |
| 2013 | 22.60 |
| 2014 | 23.88 |
| 2015 | 30.81 |
| 2016 | 31.01 |
| 2017 | 25.31 |
| 2018 | 19.98 |

From HPRCC (2019)

**Table A1.5.** Grazing acre allocation for the eight scenario possibilities for mean mature cow weight.

| Mean Mature Cow Weight, kg | Full season grazing acres allocated per pair | Full season grazing acres allocated per yearling heifer | Grazing acres allocated per post-weaning replacement heifer |
| --- | --- | --- | --- |
| 454 | 5.83 | 3.33 | 2.16 |
| 499 | 6.26 | 3.43 | 2.32 |
| 544 | 6.68 | 3.66 | 2.47 |
| 590 | 7.10 | 3.89 | 2.63 |
| 635 | 7.50 | 4.11 | 2.77 |
| 680 | 7.90 | 4.33 | 2.93 |
| 726 | 8.30 | 4.55 | 3.07 |
| 771 | 8.68 | 4.76 | 3.21 |

**Table A1.6.** Diet nutrient densities by month.

| Diet | Month | NEm, Mcal/kg | NEg, Mcal/kg | DE, Mcal/kg (used for determining calf DMI) |
| --- | --- | --- | --- | --- |
| Base (73% alfalfa, 19% wheat straw, and 8% corn) | Jan-Dec | 1.2 | 0.64 | NA |
| Supplement (60% alfalfa, 40% corn) | Jan-Dec | 1.63 | 1.02 | 3.08 |
| Bluestem Forage |  |  |  |  |
|  | Jan-Mar | 0.71 | 0.18 | 1.89 |
|  | Apr-Jun | 1.48 | 0.90 | 2.86 |
|  | Jul-Aug | 1.10 | 0.54 | 2.12 |
|  | Sep-Dec | 0.71 | 0.18 | 1.89 |

Calculated using estimates from NASEM (2016) and Kuhl et al. (1993).

**Table A1.7.** Assorted nutrition parameters.

| Model Parameter | Distribution Type | Distribution Parameters | Reference | Notes |
| --- | --- | --- | --- | --- |
| Percent of forage remaining at end of grazing season- goal | Deterministic | 40% |  |  |
| Metabolizable energy, Mcal, per kg of diet | Deterministic | 2.0 | NASEM (2016) | Assumed to be the same for all diets. Only a factor when calculating NEm requirements from gestation. |

**Table A1.8.** Daily maximum DMI as percent of SBW by MW category and animal production category.

| Animal Category | Maximum Daily DMI (percent of SBW) |
| --- | --- |
| 454 kg MW |  |
| Post-weaning Non-pregnant Replacement Heifer | 2.9 |
| Bred Yearling Heifer | 2.9 |
| Two-Year-Old Cow | 2.5 |
| Three-Year-Old Cow | 2.5 |
| Mature Cow (>= 4-Years-Old) | 2.4 |
| 499 kg MW |  |
| Post-weaning Non-pregnant Replacement Heifer | 2.9 |
| Bred Yearling Heifer | 2.7 |
| Two-Year-Old Cow | 2.4 |
| Three-Year-Old Cow | 2.4 |
| Mature Cow (>= 4-Years-Old) | 2.3 |
| 544 kg MW |  |
| Post-weaning Non-pregnant Replacement Heifer | 2.9 |
| Bred Yearling Heifer | 2.7 |
| Two-Year-Old Cow | 2.4 |
| Three-Year-Old Cow | 2.4 |
| Mature Cow (>= 4-Years-Old) | 2.3 |

**Table A1.8 (*cont*.)** Daily maximum DMI as percent of SBW by MW category and animal production category.

| Animal Category | Maximum Daily DMI (percent of SBW) |
| --- | --- |
| 590 kg MW |  |
| Post-weaning Non-pregnant Replacement Heifer | 2.7 |
| Bred Yearling Heifer | 2.7 |
| Two-Year-Old Cow | 2.3 |
| Three-Year-Old Cow | 2.3 |
| Mature Cow (>= 4-Years-Old) | 2.2 |
| 635 kg MW |  |
| Post-weaning Non-pregnant Replacement Heifer | 2.7 |
| Bred Yearling Heifer | 2.7 |
| Two-Year-Old Cow | 2.3 |
| Three-Year-Old Cow | 2.3 |
| Mature Cow (>= 4-Years-Old) | 2.2 |
| 680 kg MW |  |
| Post-weaning Non-pregnant Replacement Heifer | 2.7 |
| Bred Yearling Heifer | 2.7 |
| Two-Year-Old Cow | 2.3 |
| Three-Year-Old Cow | 2.3 |
| Mature Cow (>= 4-Years-Old) | 2.2 |

**Table A1.8 (*cont*.)** Daily maximum DMI as percent of SBW by MW category and animal production category.

| Animal Category | Maximum Daily DMI (percent of SBW) |
| --- | --- |
| 726 kg MW |  |
| Post-weaning Non-pregnant Replacement Heifer | 2.7 |
| Bred Yearling Heifer | 2.7 |
| Two-Year-Old Cow | 2.2 |
| Three-Year-Old Cow | 2.2 |
| Mature Cow (>= 4-Years-Old) | 2.1 |
| 771 kg MW |  |
| Post-weaning Non-pregnant Replacement Heifer | 2.7 |
| Bred Yearling Heifer | 2.7 |
| Two-Year-Old Cow | 2.2 |
| Three-Year-Old Cow | 2.2 |
| Mature Cow (>= 4-Years-Old) | 2.1 |

**Table A1.9.** BCS and corresponding body fat composition, percent of MSBW, and Mcal per kg of EBW loss and EBW gain.

| BCS | Percent Body Fat EBW Composition | Percent of MSBW (BCS 5) | Mcal per kg EBW Loss | Mcal per kg EBW Gain |
| --- | --- | --- | --- | --- |
| 1 | 3.77 | 71.6 | 3.69 | 4.22 |
| 2 | 7.54 | 78.7 | 4.22 | 4.76 |
| 3 | 11.30 | 85.8 | 4.76 | 5.30 |
| 4 | 15.07 | 92.9 | 5.30 | 5.84 |
| 5 | 18.89 | 100.0 | 5.84 | 6.38 |
| 6 | 22.61 | 107.1 | 6.38 | 6.91 |
| 7 | 26.38 | 114.2 | 6.91 | 7.45 |
| 8 | 30.15 | 121.3 | 7.45 | 7.99 |
| 9 | 33.91 | 128.4 | 7.99 | 8.60 |

Adapted from NASEM (2016)

**Table A1.10.** Maximum base fed diet intake by animal production category.

| Animal Production Category | Maximum Base Fed Diet Intake, kg/d |
| --- | --- |
| Nursing Calf* | 7.0 |
| Post-weaning Non-pregnant Replacement Heifer | 13.0 |
| Bred Yearling Heifer | 13.0 |
| Two-Year-Old Cow | 16.0 |
| Three-Year-Old Cow | 16.0 |
| Mature Cow (>= 4-Years-Old) | 16.0 |

*Base Fed Diet for Nursing Calves is equivalent to Supplement Diet for all other animal categories

**Table A1.11.** Reproductive cyclicity.

| Model Parameter | Distribution Type | Distribution Parameters | Reference |
| --- | --- | --- | --- |
| Postpartum Interval, d - Primiparous Cows |  |  | Ciccioli et al. (2003), Berardinelli et al. (2005), Endecott et al. (2007), and expert opinion |
| BCS 1 | Pert | (350, 350, 350) |  |
| BCS 2 | Pert | (135, 150, 165) |  |
| BCS 3 | Pert | (85, 100, 115) |  |
| BCS 4 | Pert | (65, 80, 95) |  |
| BCS 5 | Pert | (55, 70, 85) |  |
| BCS 6 | Pert | (45, 60, 75) |  |
| BCS 7 | Pert | (30, 45, 60) |  |
| BCS 8 | Pert | (30, 45, 60) |  |
| BCS 9 | Pert | (30, 45, 60) |  |

**Table A1.11 (*cont*).** Reproductive cyclicity.

| Model Parameter | Distribution Type | Distribution Parameters | Reference |
| --- | --- | --- | --- |
| PostPartum Interval, d - Multiparous Cows |  |  | Graham (1982), Rutter and Randel (1984), Houghton et al. (1990), Cushman et al. (2007), Lents et al. (2008), and expert opinion |
| BCS 1 | Pert | (350, 350, 350) |  |
| BCS 2 | Pert | (135, 150, 165) |  |
| BCS 3 | Pert | (75, 90, 105) |  |
| BCS 4 | Pert | (55, 70, 85) |  |
| BCS 5 | Pert | (45, 60, 75) |  |
| BCS 6 | Pert | (35, 50, 65) |  |
| BCS 7 | Pert | (30, 35, 50) |  |
| BCS 8 | Pert | (30, 35, 50) |  |
| BCS 9 | Pert | (30, 35, 50) |  |
| Dystocia Probability per Parturition |  |  | McDermott et al. (1990), USDA (2008), and expert opinion |
| Multiparous Cow | Normal | (0.05, 0.01, lower=0) |  |
| Primiparous Cow- Calf birth weight < 40.82 kg | Normal | (0.08, 0.01, lower=0) |  |
| Primiparous Cow- Calf birth weight >= 40.82 kg | Normal | (0.5, 0.01, lower=0) |  |

**Table A1.11 (*cont*).** Reproductive cyclicity.

| Model Parameter | Distribution Type | Distribution Parameters | Reference |
| --- | --- | --- | --- |
| Additional PPI, d, resulting from dystocia | Normal | (10, 2, lower=0) | Doornbos et al. (1984), Bellows et al. (1988), and expert opinion. |
| Pregnancy probability at d equal to estrous cycle length after breeding |  |  | Spell et al. (2001), Chagas et al. (2002), Aherin et al. (2018), and expert opinion |
| Heifers | Normal | (0.71, 0.01, upper = 0.8) | Cundiff et al. (1974) |
| Primiparous Cows | Normal | (0.61, 0.01, upper=0.8) | Cundiff et al. (1974) |
| Multiparous Cows | Normal | (0.71, 0.01, upper = 0.8) | Cundiff et al. (1974) |

**Table A1.11 (*cont*).** Reproductive cyclicity.

| Model Parameter | Distribution Type | Distribution Parameters | Reference |
| --- | --- | --- | --- |
| Daily mean probability of returning to cyclicity after establishing pregnancy |  |  |  |
| d 25 to d 45 | Normal | (0.002, 0.0002, lower = 0) | Whittier et al. (1991), Lamb et al. (2008), Aherin et al. (2018), and expert opinion. |
| d 46 to d 65 | Normal | (0.0005, 0.00002, lower = 0) | Whittier et al. (1991), Lamb et al. (2008), Aherin et al. (2018), and expert opinion. |
| d > 65 | Normal | (0.0001, 0.00002, lower = 0) | Dziuk and Bellows (1983), van Wagtendonk-de Leeuw et al. (2000), Aherin et al. (2018), and expert opinion |
| Individual Gestation length | Normal | (285, 7) | Expert opinion |

**Table A1.12.** Culling.

| Model Parameter | Distribution Type | Distribution Parameters | Reference |
| --- | --- | --- | --- |
| Pregnancy determination (days after breeding season end) | Deterministic | 60 | User-defined |
| Age, d, of oldest calf at weaning | Deterministic | 220 | User-defined |
| Maximum cow age | Deterministic | 13 | User-defined |
| Minimum culling percentage by cow age, yr (involuntary and voluntary combined) |  | culls within age/exposed within age, percent | Wittum et al.(1994), USDA (2010), Cushman et al. (2013), Ringwall (2014), and expert opinion |
| 1 | Deterministic | 5 |  |
| 2 | Deterministic | 10 |  |
| 3 | Deterministic | 6 |  |
| 4 | Deterministic | 6 |  |
| 5 | Deterministic | 6 |  |
| 6 | Deterministic | 6 |  |
| 7 | Deterministic | 6 |  |
| 8 | Deterministic | 6 |  |
| 9 | Deterministic | 8 |  |
| 10 | Deterministic | 10 |  |
| 11 | Deterministic | 40 |  |
| 12 | Deterministic | 50 |  |
| 13 | Deterministic | 100 |  |

**Table A1.13.** Morbidity and mortality.

| Model Parameter | Distribution Type | Distribution Parameters | Reference |
| --- | --- | --- | --- |
| Daily probability of preweaned calf morbidity |  |  | Wittum et al. (1994), Sanderson and Dargatz (2000), USDA (2010), and expert opinion |
| Dystocia and neonatal period (d 1-3 after parturition) | Normal | (0.01, 0.005, lower=0) |  |
| No dystocia and neonatal period (d 1-3 after parturition) | Normal | (0.005, 0.001, lower=0) |  |
| Dystocia and post-neonatal period to weaning | Normal | (0.0004, 0.00001, lower=0) |  |
| No dystocia and post-neonatal period to weaning | Normal | (0.0002, 0.00001, lower=0) |  |

**Table A1.13 (*cont*).** Morbidity and mortality.

| Model Parameter | Distribution Type | Distribution Parameters | Reference |
| --- | --- | --- | --- |
| Daily probability of preweaned calf mortality |  |  | Laster and Gregory (1973), Patterson et al. (1987), Wittum et al. (1994), USDA (2010), and expert opinion |
| Dystocia, no morbidity, and neonatal period | Normal | (0.06, 0.005, lower=0) |  |
| Dystocia, morbidity, and neonatal period | Normal | (0.1, 0.0005, lower=0) |  |
| No dystocia, no morbidity, and neonatal period | Normal | (0.01, 0.001, lower=0) |  |
| No dystocia, morbidity, and neonatal period | Normal | (0.05, 0.001, lower=0) |  |
| Dystocia, no morbidity, and post-neonatal period to weaning | Normal | (0.0001, 0.00001, lower=0) |  |
| Dystocia, morbidity, and post-neonatal period to weaning | Normal | (0.001, 0.0001, lower=0) |  |
| No dystocia, no morbidity, and post-neonatal period to weaning | Normal | (0.0001, 0.00001, lower=0) |  |
| No dystocia, morbidity, and post-neonatal period to weaning | Normal | (0.0005, 0.0001, lower=0) |  |

**Table A1.13 (*cont*).** Morbidity and mortality.

| Model Parameter | Distribution Type | Distribution Parameters | Reference |
| --- | --- | --- | --- |
| Daily probability of postweaning mortality |  |  | USDA (2010), and expert opinion |
| Dystocia at birth | Normal | (0.00005, 0.00001,lower=0) |  |
| No Dystocia at birth | Normal | (0.000025, 0.00001,lower=0) |  |
| Daily probability of mature mortality | Normal | (0.000025, 0.00001,lower=0) | USDA (2010), and expert opinion |
| Percent reduction in WW from morbidity | Normal | (0.065, 0.0065) | Wittum et al. (1994) |

**Table A1.14.** Calf growth**.**

| Model Parameter | Distribution Type | Distribution Parameters | Reference |
| --- | --- | --- | --- |
| Calf birth weights |  |  |  |
| Bull calf, two-year-old dam mean birth weight adjustment, kg | Deterministic | -3.63 | BIF (2010) |
| Bull calf, three-year-old dam mean birth weight adjustment, kg | Deterministic | -2.27 | BIF (2010) |
| Bull calf, four-year-old dam mean birth weight adjustment, kg | Deterministic | -0.91 | BIF (2010) |
| Bull calf, eleven-year-old and older dam mean birth weight adjustment, kg | Deterministic | -1.36 | BIF (2010) |

**Table A1.14 (*cont*).** Calf growth**.**

| Model Parameter | Distribution Type | Distribution Parameters | Reference |
| --- | --- | --- | --- |
| Calf birth weights |  |  |  |
| Heifer calf, two-year-old dam mean birth weight adjustment, kg | Deterministic | -3.17 | BIF (2010) |
| Heifer calf, three-year-old dam mean birth weight adjustment, kg | Deterministic | -2.27 | BIF (2010) |
| Heifer calf, four-year-old dam mean birth weight adjustment, kg | Deterministic | -0.91 | BIF (2010) |
| Heifer calf, eleven-year-old and older dam mean birth weight adjustment, kg | Deterministic | -1.36 | BIF (2010) |

**Literature Cited**

Aherin, D. G., J. M. Bormann, J. L. Heier Stamm, M. D. MacNeil, and R. L. Weaber. 2018. Decision-making tools: stochastic simulation model accounting for the impacts of biological variation on success of bovine embryo transfer programs. Translational Animal Science. 2:451-462. doi:[10.1093/tas/txy087](C:\\Users\\Dr. Heier Stamm\\Documents\\GRADUA~2\\PHDTHE~1\\AHERIN~1\\JOURNA~1\\10.1093\\tas\\txy087)

American Angus Association (AAA). 2019b. Genetic trend EPD/$Value by birth year. Accessed 10 December 2019. <https://www.angus.org/Nce/GeneticTrends.aspx>

American Angus Association (AAA). 2019c. Heritabilities. Accessed 10 December 2019. <https://www.angus.org/Nce/Heritabilities.aspx>

Beef Improvement Federation (BIF). 2010. BIF guidelines for uniform beef improvement programs. 9th ed. Beef Improvement Federation, North Mississippi Research and Extension Center, Verona, MS. <https://beefimprovement.org/wp-content/uploads/2018/03/BIFGuidelinesFinal_updated0318.pdf>

Bellows, R. A., R. E. Short, R. B. Staigmiller, and W. L. Milmine. 1988. Effects of induced parturition and early obstetrical assistance in beef cattle. J. Anim. Sci. 66:1073-1080. doi: [10.2527/jas1988.6651073x](https://doi.org/10.2527/jas1988.6651073x)

Berardinelli, J. G., P. S., Joshi, and S. A. Tauck. 2005. Postpartum resumption of ovarian cycling activity in first-calf suckled beef cows exposed to familiar or unfamiliar bulls. Anim. Reprod. Sci. 90:201-209. doi:[10.1016/j.anireprosci.2005.02.005](file:///C:\Users\Dr.%20Heier%20Stamm\Documents\Graduate%20Student%20Advising\PhD%20Thesis%20Committees\Aherin,%20Dustin\Journal%20of%20Animal%20Science%20Paper%201\10.1016\j.anireprosci.2005.02.005)

Chagas e Silva, J., L. Lopes da Costa, and J. Robalo Silva. 2002. Plasma progesterone profiles and factors affecting embryo-fetal mortality following embryo transfer in dairy cattle. Theriogenology*.* 58:51–59. doi: [10.1016/S0093-691X(02)00906-8](https://doi.org/10.1016/S0093-691X(02)00906-8)

Ciccioli, N. H., R. P. Wettemann, L. J. Spicer, C. A. Lents, F. J. White, and D. H. Kesler. 2003. Influence of body condition at calving and postpartum nutrition on endocrine function and reproductive performance of primiparous beef cows. J. Anim. Sci*.* 81:3107-3120. doi:[10.2527/2003.81123107x](file:///C:\Users\Dr.%20Heier%20Stamm\Documents\Graduate%20Student%20Advising\PhD%20Thesis%20Committees\Aherin,%20Dustin\Journal%20of%20Animal%20Science%20Paper%201\10.2527\2003.81123107x)

Cundiff, L. V., K. E. Gregory, and R. M. Koch. 1974. Effects of heterosis on reproduction in Herford, Angus and Shorthorn cattle. J. Anim. Sci. 38:711–727. doi:10.2527/jas1974.384711x.

Cushman, R. A., L. K. Kill, R. N. Funston, E. M. Mousel, and G. A. Perry. 2013. Heifer calving date positively influences calf weaning weights through six parturitions. J. Anim. Sci. 91:4486-4491. doi:[10.2527/jas.2013-6465](file:///C:\Users\Dr.%20Heier%20Stamm\Documents\Graduate%20Student%20Advising\PhD%20Thesis%20Committees\Aherin,%20Dustin\Journal%20of%20Animal%20Science%20Paper%201\10.2527\jas.2013-6465)

Cushman, R. A., M. F. Allen, R. M. Thallman, and L. V. Cundiff. 2007. Characterization of biological types of cattle (Cycle VII): Influence of postpartum interval and estrous cycle length on fertility. J. Anim. Sci. 85:2156-2162. doi:[10.2527/jas.2007-0136](file:///C:\Users\Dr.%20Heier%20Stamm\Documents\Graduate%20Student%20Advising\PhD%20Thesis%20Committees\Aherin,%20Dustin\Journal%20of%20Animal%20Science%20Paper%201\10.2527\jas.2007-0136)

Doornbos, D. E., R. A. Bellows, P. J. Burfening, and B. W. Knapp. 1984. Effects of dam age, prepartum nutrition and duration of labor on productivity and postpartum reproduction in beef females. J. Anim. Sci. 59:1-10. doi:[10.2527/jas1984.5911](file:///C:\Users\Dr.%20Heier%20Stamm\Documents\Graduate%20Student%20Advising\PhD%20Thesis%20Committees\Aherin,%20Dustin\Journal%20of%20Animal%20Science%20Paper%201\10.2527\jas1984.5911)

Dziuk, P. J., and R. A. Bellows. 1983. Management of reproduction of beef cattle, sheep and pigs. J. Anim. Sci*.* 57:355-379. doi: [10.2527/animalsci1983.57Supplement_2355x](https://doi.org/10.2527/animalsci1983.57Supplement_2355x)

Endecott, R. L., S. H. Cox, and M. K. Petersen. 2007. Impacts of supplemental glucogenic precursors and cow age on postpartum range cow performance. Western Section, American Society of Animal Science 58:352-357.

Graham, J. 1982. The effect of body condition of beef cows at calving and post calving nutrition on calf growth rate and cow fertility. Proc. Aust. Soc. Anim. Prod. 14:309–312

Gregory, K. E., L. V. Cundiff, and R. M. Koch. 1995. Genetic and phenotypic (co)variances for production traits of intact male populations of purebred and composite beef cattle. J. Anim. Sci. 73:2227–2234. doi:10.2527/1995.7382227x.

High Plains Regional Climate Center (HPRCC). Manhattan Agronomy Farm. Accessed 15 November 2019. <https://hprcc.unl.edu/index.php>

Houghton, P. L., R. P. Lemenager, L.A. Horstman, K.S. Hendrix, and G.E. Moss. 1990. Effects of body composition, pre- and postpartum energy level and early weaning on reproductive performance of beef cows and preweaning calf gain. J. Anim. Sci. 68:1438-1446. doi:[10.2527/1990.6851438x](file:///C:\Users\Dr.%20Heier%20Stamm\Documents\Graduate%20Student%20Advising\PhD%20Thesis%20Committees\Aherin,%20Dustin\Journal%20of%20Animal%20Science%20Paper%201\10.2527\1990.6851438x)

Kuhl, G., D. Simms, and C. Bandyk. 1993. Nutritional composition of feedstuffs for beef cattle. Kansas State University Agricultural Experiment Station and Cooperative Extension Service. Bulletin L-884.

Lamb, G. C., C. R. Dahlen, K. A. Vonnahme, G. R. Hansen, J. D. Arseneau, G. A. Perry, R. S. Walker, J. Clement, and J. D. Arthington. 2008. Influence of a CIDR prior to bull breeding on pregnancy rates and subsequent calving distribution. Anim. Reprod. Sci. 108:269–278. doi:[10.1016/j.anireprosci.2007.08.012](file:///C:\Users\Dr.%20Heier%20Stamm\Documents\Graduate%20Student%20Advising\PhD%20Thesis%20Committees\Aherin,%20Dustin\Journal%20of%20Animal%20Science%20Paper%201\10.1016\j.anireprosci.2007.08.012)

Laster, D. B., and K. E. Gregory. 1973. Factors influencing peri- and early postnatal Ccalf mortality. J. Anim. Sci. 37:1092-1097. doi:[10.2527/jas1973.3751092x](file:///C:\Users\Dr.%20Heier%20Stamm\Documents\Graduate%20Student%20Advising\PhD%20Thesis%20Committees\Aherin,%20Dustin\Journal%20of%20Animal%20Science%20Paper%201\10.2527\jas1973.3751092x)

Lents, C. A., F. J. White, N. H. Ciccioli, R. P. Wettemann, L. J. Spicer, and D. L. Lalman. 2008. Effects of body condition score at parturition and postpartum protein supplementation on estrous behavior and size of the dominant follicle in beef cows. J. Anim. Sci. 86:2549-2556. doi:[10.2527/jas.2008-1114](file:///C:\Users\Dr.%20Heier%20Stamm\Documents\Graduate%20Student%20Advising\PhD%20Thesis%20Committees\Aherin,%20Dustin\Journal%20of%20Animal%20Science%20Paper%201\10.2527\jas.2008-1114)

McDermott J. J., O. B. Allen, S. W. Martin, and D. M. Alves. 1992. Patterns of stillbirth and dystocia in Ontario cow-calf herds. Can J Vet Res. 56:47–55. PMID: 1586893

Morris, C. A. and J. W. Wilton. 1976. Influence of body size on the biological efficiency of cows: a review. Can. J. Anim. Sci. 56:613-647. doi:10.4141/cjas76-076.

NASEM. 2016. Nutrient requirements of beef cattle. 8^th^ ed. Washington, DC The National Academies Press. doi:[10.17226/19014](file:///C:\Users\Dr.%20Heier%20Stamm\Documents\GRADUA~2\PHDTHE~1\AHERIN~1\JOURNA~1\10.17226\19014)

Patterson, D. J., R. A. Bellows, P. J. Burfening, and J. B. Carr. 1987. Occurrence of neonatal and postnatal mortality in range beef cattle. I. Calf loss incidence from birth to weaning, backward and breech presentations and effects of calf loss on subsequent pregnancy rate of dams. Theriogenology. 28: 557-571. doi: 10.1016/0093-691x(87)90273-1.

Ringwall, K. 2014. Age and weight are cow herd dynamics. BeefTalk. <https://www.ag.ndsu.edu/news/columns/beeftalk/beeftalk-age-and-weight-are-cow-herd-dynamics/>

Sanderson, M. W., and D. A. Dargatz. 2000. Risk factors for high herd level calf morbidity risk from birth to weaning in beef herds in the USA. Prev. Vet. Med. 44:97-106. doi:[10.1016/S0167-5877(99)00112-9](file:///C:\Users\Dr.%20Heier%20Stamm\Documents\Graduate%20Student%20Advising\PhD%20Thesis%20Committees\Aherin,%20Dustin\Journal%20of%20Animal%20Science%20Paper%201\10.1016\S0167-5877(99)00112-9)

Spell, A. R., W. E. Beal, L. R. Corah, and G. C. Lamb. 2001. Evaluating recipient and embryo factors that affect pregnancy rates of embryo transfer in beef cattle. Theriogenology. 56:287-297. doi: [10.1016/S0093-691X(01)00563-5](https://doi.org/10.1016/S0093-691X(01)00563-5)Tedeschi, L. O., and D. G. Fox. 2009. Predicting milk and forage intake of nursing calves. J. Anim. Sci. 87:3380–3391. doi:10.2527/jas.2009-2014.

USDA. 2008. Beef 2007–08, Part III: Changes in the U.S. Beef Cow-Calf Industry, 1993–2008. USDA:APHIS:VS, CEAH. Fort Collins, CO. #518.0509

USDA. 2010. Beef 2007–08, Part IV: Reference of Beef Cow-calf Management Practices in the United States, 2007–08. USDA:APHIS:VS, CEAH. Fort Collins, CO. #523.0210

van Wagtendonk-de Leeuw, A. M., E. Mullaart, A. P. W. de Roos, J. S. Merton, J. H. G. den Daas, B. Kemp, and L. de Ruigh. 2000. Effects of different reproduction techniques: AI, MOET, or IVP, on health and welfare of bovine offspring. Theriogenology. 53:575–597. doi:[10.1016/S0093-691X(99)00259-9](file:///C:\Users\Dr.%20Heier%20Stamm\Documents\Graduate%20Student%20Advising\PhD%20Thesis%20Committees\Aherin,%20Dustin\Journal%20of%20Animal%20Science%20Paper%201\10.1016\S0093-691X(99)00259-9)

Whittier, J. C., R. W. Caldwell, R. V. Anthony, M.F. Smith, and R.E. Morrow. 1991. Effect of a prostaglandin F2α injection 96 hours after introduction of intact bulls on estrus and calving distribution of beef cows. J. Anim. Sci. 69:4670-4677. doi:[10.2527/1991.69124670x](file:///C:\Users\Dr.%20Heier%20Stamm\Documents\Graduate%20Student%20Advising\PhD%20Thesis%20Committees\Aherin,%20Dustin\Journal%20of%20Animal%20Science%20Paper%201\10.2527\1991.69124670x)

Wittum, T. E., M.D. Salman, M. E. King, R. G. Mortimer, K. G. Odde, and D. L. Morris. 1994. The influence of neonatal health on weaning weight of Colorado, USA beef calves. Prev. Vet. Med. 19:15-25. doi:[10.1016/0167-5877(94)90011-6](file:///C:\Users\Dr.%20Heier%20Stamm\Documents\Graduate%20Student%20Advising\PhD%20Thesis%20Committees\Aherin,%20Dustin\Journal%20of%20Animal%20Science%20Paper%201\10.1016\0167-5877(94)90011-6)
