# Supplementary figures and images for: Stochastic, individual animal systems simulation model of beef cow–calf production: development and validation
Source: Transl Anim Sci. 2022 Dec 3;7(1):txac155. doi: 10.1093/tas/txac155 (PMC9930734; doi:10.1093/tas/txac155)

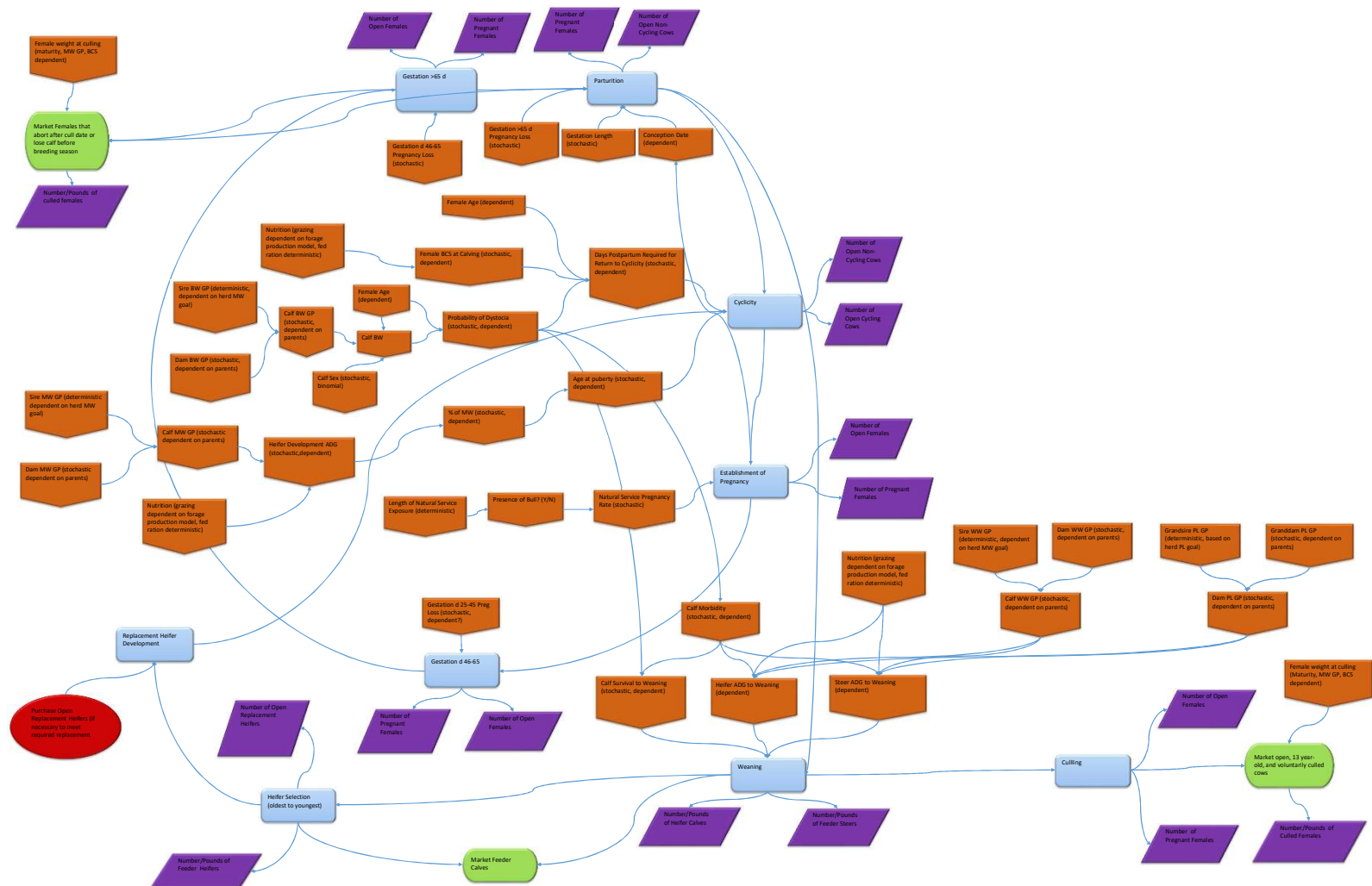

Supplement: txac155_suppl_Supplementary_Figure_S1 [file txac155_suppl_supplementary_figure_s1.pdf]
